# Metal–organic frameworks as O_2_-selective adsorbents for air separations†

**DOI:** 10.1039/d2sc03577d

**Published:** 2022-08-11

**Authors:** David E. Jaramillo, Adam Jaffe, Benjamin E. R. Snyder, Alex Smith, Eric Taw, Rachel C. Rohde, Matthew N. Dods, William DeSnoo, Katie R. Meihaus, T. David Harris, Jeffrey B. Neaton, Jeffrey R. Long

**Affiliations:** Department of Chemistry, University of California Berkeley Berkeley California 94720 USA jrlong@berkeley.edu; Department of Physics, University of California Berkeley Berkeley California 94720 USA; Department of Chemical and Biomolecular Engineering, University of California Berkeley Berkeley California 94720 USA; Materials Science Division, Lawrence Berkeley National Laboratory Berkeley California 94720 USA; Molecular Foundry, Lawrence Berkeley National Laboratory Berkeley California 94720 USA; Kavli Nanosciences Institute at Berkeley Berkeley California 94720 USA

## Abstract

Oxygen is a critical gas in numerous industries and is produced globally on a gigatonne scale, primarily through energy-intensive cryogenic distillation of air. The realization of large-scale adsorption-based air separations could enable a significant reduction in associated worldwide energy consumption and would constitute an important component of broader efforts to combat climate change. Certain small-scale air separations are carried out using N_2_-selective adsorbents, although the low capacities, poor selectivities, and high regeneration energies associated with these materials limit the extent of their usage. In contrast, the realization of O_2_-selective adsorbents may facilitate more widespread adoption of adsorptive air separations, which could enable the decentralization of O_2_ production and utilization and advance new uses for O_2_. Here, we present a detailed evaluation of the potential of metal–organic frameworks (MOFs) to serve as O_2_-selective adsorbents for air separations. Drawing insights from biological and molecular systems that selectively bind O_2_, we survey the field of O_2_-selective MOFs, highlighting progress and identifying promising areas for future exploration. As a guide for further research, the importance of moving beyond the traditional evaluation of O_2_ adsorption enthalpy, Δ*H*, is emphasized, and the free energy of O_2_ adsorption, Δ*G*, is discussed as the key metric for understanding and predicting MOF performance under practical conditions. Based on a proof-of-concept assessment of O_2_ binding carried out for eight different MOFs using experimentally derived capacities and thermodynamic parameters, we identify two existing materials and one proposed framework with nearly optimal Δ*G* values for operation under user-defined conditions. While enhancements are still needed in other material properties, the insights from the assessments herein serve as a guide for future materials design and evaluation. Computational approaches based on density functional theory with periodic boundary conditions are also discussed as complementary to experimental efforts, and new predictions enable identification of additional promising MOF systems for investigation.

## Abstract 2

This Perspective summarizes progress in the development of O_2_-selective metal–organic frameworks for adsorptive air separations and identifies key metrics and design considerations toward optimizing material performance for practical applications.

## Graphical abstract

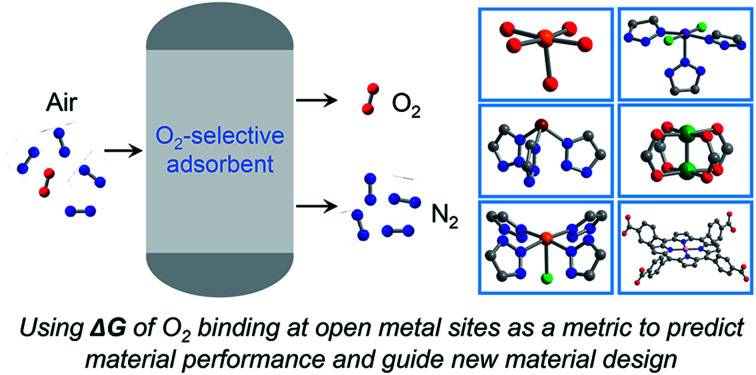

## 1. Introduction

### 1.1 Current air separation technologies and their limitations

Oxygen is one of the foundational gases of the industrial sector.^1^ Today, fossil fuel combustion alone consumes O_2_ directly from air on a massive scale of ∼25 gigatonnes per year, about 8 gigatonnes more than the annual natural production of O_2_.^2^ Moreover, enriched (>21%) or high-purity oxygen (>95%) is essential for numerous applications, including within the medical and aerospace industries,^1,3^ as well as in a variety of chemical processes, such as the production of phthalic anhydride, acetaldehyde synthesis *via* the Wacker Process, and ethylene oxide production.^1,4^ The COVID-19 pandemic has also highlighted the importance of medical-grade oxygen and its existing supply chain issues.^5^ These and many other processes depend on the separation of oxygen from air. As a result, this process is performed on a scale of hundreds of millions of tonnes per year.^6^

Importantly, high-purity O_2_ is also critical for next-generation carbon capture processes poised to play a critical role in reducing emissions from large-scale combustion plants. For example, oxy-fuel power plants, which are currently in the pilot stage, use O_2_ (∼95%) instead of air to produce a concentrated CO_2_ flue gas stream.^7–9^ This process renders post-combustion CO_2_ capture more viable by reducing the energy needed for regeneration while also decreasing NO_*x*_ emissions. Likewise, high-purity O_2_ is necessary for carrying out pre-combustion CO_2_ capture in fossil fuel gasification plants.^10,11^

Approximately 70% of O_2_ generated in industry is obtained *via* cryogenic distillation of air,^12^ which is predominantly a centralized process that takes place in large air separation plants. First pioneered by Linde in the late 19^th^ century, this process exploits the small difference in the boiling points of O_2_ and N_2_ (Table 1).^1,13^ In brief, low-temperature air is fed into large distillation columns that feature pressure and temperature gradients and numerous trays where liquid and vapor phases equilibrate. Within these columns, nitrogen vapor rises and increases in purity, while liquid oxygen descends the columns and increases in purity. Cryogenic distillation plants can generate O_2_ with 95% purity or higher at 1000 to 5000 tonnes per day. This method is a remarkable feat of engineering, considering the small difference in vapor pressure between the two gases. Nevertheless, cryogenic separation units are very capital-intensive projects that require operation at relatively high capacities.^14^ As a result, the energy required to separate one tonne of O_2_ from air is approximately 245 kW h (Table 2),^6,14^ more than four times greater than the minimum separation work of 58 kW h per tonne at 25 °C (see ESI Section S1†).^14^ Further, owing primarily to the similar vapor pressures of argon and O_2_,^13^ achieving O_2_ purities greater than 95% requires additional expensive refrigeration that increases the overall energy demand per tonne of O_2_.^6^

**Table 1:** Relevant properties of the key components of air

	N_2_	O_2_	Ar
Molar fraction	0.78	0.21	00.93
Normal boiling point (K)	77.3	90.2	87.3
Kinetic diameter (Å)	3.64	3.47	3.54
Polarizability × 10^25^ (cm^3^)	17.703	15.812	16.411
Quadrupole moment × 10^26^ (esu cm^2^)	1.52	0.39	0
Electron affinity (eV)	<0	0.450	<0

**Table 2:** Overview of current air separation processes

	Cryogenic distillation^a^	Adsorptive separations^b^	Membrane separations^c^
Operating pressures	1–6 bar	0.2–6 bar	1–6 bar
Operating temperatures	77–298 K	298–318 K	298–318 K
O_2_ purity achieved	>95%	<95%	<50%
O_2_ production scale per day	1000 tonnes	100 tonnes	10 tonnes
Largest reported capacity per day	5000 tonnes (Air Liquide)	340 tonnes (Linde)	25 tonnes
Energy demand per tonne	220–270 kW h at 95% purity	500 kW h at 95% purity	300 kW h at 40% purity

a Values obtained from ref. 6, 14, and 19.

b Values obtained from ref. 6, 14, and 19.

c Values obtained from ref. 6 and 20. The largest reported capacity per day is an estimate from ref. 6.

Adsorptive air separation represents the second most common process for oxygen production, accounting for ∼20% of O_2_ produced in industry.^14^ This approach currently exploits differences in the polarizability and quadrupole moment of O_2_ and N_2_ (Table 1). Briefly, pressurized air is fed through beds containing an adsorbent that selectively binds N_2_. The most commonly used adsorbent is a low-silica zeolite exchanged with lithium ions, known as Li-LSX,^12,15^ which features exposed Li^+^ sites that preferentially interact with N_2_. Once the adsorbent is saturated with the gas, the bed is depressurized to near ambient pressure, resulting in elution of an O_2_-enriched stream, followed by N_2_. A process operating under such conditions is referred to as pressure swing adsorption (PSA). Air can also be fed into the adsorbent bed at near ambient pressure, and in this case vacuum or heat can then be applied to regenerate the adsorbent, in so-called vacuum swing adsorption (VSA) or temperature swing adsorption (TSA) processes.^12,14,16,17^ Further, the above separation methods can be combined to engender pressure-temperature or vacuum-temperature swing adsorption processes. Importantly, adsorptive air separations can serve a complementary role to cryogenic distillation, as they can operate under a more variable load (for instance, by changing the number of adsorbent beds in use), are substantially less capital-intensive for small-scale applications, and entail minimal startup time. Adsorptive air separation plants typically consume 500 kW h per tonne of O_2_ produced at 90–95% purity,^14,18^ although a much lower value of 270 kW h per tonne of O_2_ has been reported (Table 2).^12^ This energy demand is nearly double that of cryogenic distillation, and the resulting oxygen purity is generally lower. This higher energy cost arises due to the need to regenerate the adsorbent, as discussed above. Additionally, zeolites typically exhibit low N_2_ uptake, and therefore substantial quantities of adsorbent material are required—roughly 1 tonne of zeolite is needed for producing 1 tonne of O_2_ per day. In all, these shortcomings preclude the more wide-spread application of Li-LSX adsorbents for air separations.

Polymeric membrane-based air separations are the youngest commercial technology for extracting O_2_ from air, and operate by passing an air stream through a series of membrane units.^18–20^ These processes function under a solution-diffusion mechanism and discriminate oxygen and nitrogen based on their permeabilities. Industrially relevant membranes are permselective with respect to oxygen, and so the resulting permeate is O_2_-enriched air. As a result of their mechanism of operation, membrane separations can be carried out continuously under ambient conditions, without the need for regeneration. As such, polymer membranes have the potential to offer considerable reductions in capital costs and operating expenses relative to adsorption and cryogenic distillation. However, for the majority of membranes studied to date, permeate O_2_ concentrations are limited by the minor solubilization difference between O_2_ and N_2_.^21^ This shortcoming, combined with similarities in the kinetic diameters of N_2_ and O_2_, manifests as a permeability-selectivity tradeoff that limits permeate purities to below 40–50% O_2_.^18,21^ Note that ceramic membranes, which function through a different mechanism, may find use in industrial applications. However, these membranes typically require very high temperatures to operate, in the range of 800−900 °C.^19,22^

### 1.2 The potential impact of O_2_-selective adsorbents in air separations

In general, established air separation technologies operate by distinguishing O_2_ and N_2_ based on relatively small differences in their boiling points, polarizabilities, and quadrupole moments. However, the property that most distinguishes O_2_ from N_2_ (and Ar) is its electron affinity (Table 1). Oxygen can readily accept one or two electrons from a single metal center, whereas nitrogen is generally redox-inactive.^23^ Biological systems exploit this difference to selectively and reversibly bind O_2_ using iron(ii)- and copper(i)-containing proteins. In particular, hemoglobin and myoglobin,^24,25^ hemerythrin,^26^ and hemocyanin^27^ are representative examples of proteins containing metal sites that bind O_2_*via* electron transfer.

The realization of adsorbents that are capable of similar strong yet reversible chemisorption of O_2_ could transform the air separation industry. Indeed, because the partial pressure of O_2_ in air is approximately four times less than that of N_2_, the amount of O_2_-selective adsorbent needed to process a given quantity of air will be one quarter of the quantity needed for an analogous N_2_-selective adsorbent. As a result, even in a scenario where the enthalpy of O_2_ binding in an O_2_-selective adsorbent is comparable to the N_2_ binding enthalpy in Li-LSX, the overall regeneration energy needed for the O_2_-selective material would be substantially lower, perhaps even 50% lower, assuming the material exhibits a high selectivity for O_2_ (ESI Section S2†).^28^ In principle, energy-efficient O_2_-selective adsorbents could be implemented in all air separation applications that currently utilize N_2_-selective adsorbents. Additionally, O_2_-selective adsorbents could promote the adoption of new separation processes based on adsorption, including small-scale, variable-load oxygen production, mixed-matrix membrane air separations, and hybrid processes with cryogenic distillation. Overall, a commercially viable O_2_-selective adsorbent could support the decentralization of oxygen production and utilization. Furthermore, O_2_-selective adsorbents with high volumetric capacities might be less costly than cryogenic distillation for mid- and even large-scale air separations, enabling the use of smaller contactors and thereby incurring lower capital expenses.^29^

### 1.3 Metal–organic frameworks as O_2_-selective adsorbents

Research focused on the development of O_2_-selective adsorbents has been ongoing since the 1970s,^30–34^ although few materials have been discovered that show thermodynamic selectivity for oxygen over nitrogen, and no O_2_-selective adsorbent is commercially viable at present. Extensive early work focused on the development of molecular compounds that bind O_2_ in solution and in the solid-state, most notably featuring cobalt(ii).^30–34^ However, many of these complexes exhibit poor stabilities, low capacities, and/or irreversible O_2_ binding, making them unsuitable for implementation in practical air separation processes. Immobilization of cobalt complexes on porous solid substrates, such as silicates and polymers,^35–37^ has been shown to improve cycling performance, but these materials typically exhibit low O_2_ capacities. Considering these shortcomings, an ideal adsorbent would incorporate a high density of redox-active open metal sites immobilized in a robust, rigid porous framework.

Metal–organic frameworks (MOFs) can satisfy both criteria. These crystalline, porous materials are assembled from metal nodes and multitopic organic linkers, and they have emerged as promising candidates to replace traditional adsorbents in numerous industrial applications.^38,39^ Structure types of MOFs span a vast library, and in some cases, the as-synthesized material features metal ions with coordination spheres that are completed with solvent molecules introduced during synthesis. Removal of this solvent with heating under reduced pressure (desolvation) generates coordinatively-unsaturated (or “open”) metal centers that can directly interact with adsorbate molecules. The appropriate choice of metal and linker can give rise to a material featuring open metal sites that preferentially bind certain guests based on specific selectivity handles, including gas polarizability,^40^ π-acidity,^41^ and electron affinity.^42^

In 2010, the metal–organic framework Cr_3_(btc)_2_ (btc^3−^ = 1,3,5-benzenetricarboxylate) was reported to strongly bind O_2_*via* electron transfer to form a Cr^III^–O_2_^−^ adduct.^42^ The pristine material features a high-density of exposed chromium(ii) sites and accordingly displays a high O_2_ uptake of 11 wt% at 298 K and an O_2_/N_2_ selectivity of ∼22 based on single-component adsorption data, notably five times higher than that achieved with cobalt(ii) molecules immobilized on silica.^36^ This discovery represented a significant advance in the design of O_2_-selective adsorbents and validated the strategy of using redox-active open metal sites in MOFs to target guests based on differences in electron affinity. However, because of the highly exothermic O_2_ binding in Cr_3_(btc)_2_, the material capacity decreases with repeated cycling, likely owing to incomplete regeneration of the open metal sites and/or sample degradation. This result therefore also underscores the importance of designing a material exhibiting selective and reversible O_2_ binding. Since this seminal work, several studies have focused on the design of MOFs featuring open metal sites that are capable of selective oxygen capture *via* electron transfer chemistry.^43–45^ This effort has focused on the chemical space containing monovalent, divalent, and trivalent redox-active metal sites in diverse coordination environments, ranging from anionic oxygen donors in a square pyramidal geometry to nitrogen heterocycles in a trigonal pyramidal geometry.^42–48^ Although no MOF has been discovered to date that can be considered a commercially viable O_2_-selective adsorbent, a number of promising systems and materials design strategies have emerged in recent years.

In this Perspective, we provide a comprehensive survey of the field of O_2_-selective MOFs with the goal of motivating continued progress and new directions in this emerging area of research. A summary of key molecular and biological systems that reversibly bind O_2_ is presented in Section 2 to introduce the various species known to form upon binding and reduction of O_2_ at open metal sites, as well as relevant design considerations for tuning O_2_ binding in MOFs. In Section 3, these concepts are applied in a survey of key framework materials studied to date, which highlights progress and promising areas for future exploration. A critical contribution in the latter section is an in-depth examination of the relevant thermodynamic parameters and working conditions for adsorptive air separation processes. Traditionally, only O_2_ binding enthalpies have been presented in the literature along with O_2_/N_2_ selectivities at a range of temperatures, precluding meaningful comparisons across materials. We discuss the free energy of O_2_ binding as the most important metric to consider when evaluating and designing new MOFs for practical air separations and calculate Δ*G* values for preliminary comparison and benchmarking of existing materials. In Section 4, density functional theory (DFT) approaches for materials evaluation are discussed that can support experimental efforts, and promising MOF targets based on the aforementioned thermodynamic considerations are identified. A brief summary and conclusions are presented in Section 5.

## 2. Design inspiration from molecular and biological examples of reversible O_2_ binding

### 2.1 Fundamental considerations

Oxygen binding at a redox-active metal center is an exothermic reaction, facilitated by a strong charge-transfer component. The resulting enthalpic driving force is offset to varying degrees by an entropic penalty.^49,50^ The entropy of O_2_ binding is made up of translational, rotational, vibrational, and electronic components (ESI Section S3†), and ultimately cannot exceed the entropy of free O_2_.^51^ Typically, the greater the degree of charge transfer, the stronger the metal–oxygen bonding, and the fewer degrees of freedom for the bound O_2_. As such, enthalpy and entropy tend to correlate broadly.^49^ Nevertheless, as discussed below, it is important to quantify Δ*S* in addition to Δ*H*, because small entropic changes can meaningfully impact Δ*G* under practical conditions for O_2_ separations.

**Fig. 1:**
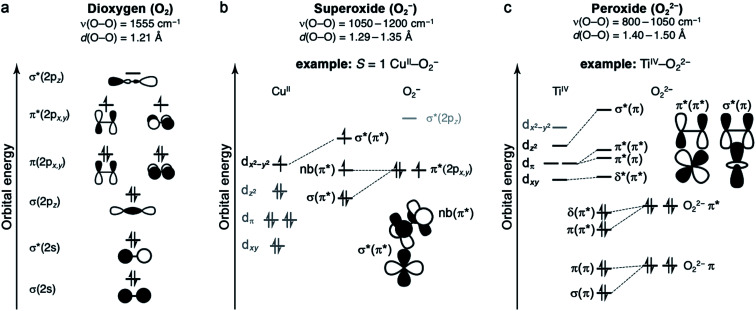
(a) Qualitative molecular orbital diagram of dioxygen. (b) Qualitative electronic structure of an *S* = 1 end-on Cu^II^–O_2_^−^ complex, with key orbital interactions highlighted. (c) Qualitative electronic structure of a side-on Ti^IV^–O_2_^2−^ complex, showing two molecular orbital interactions. For the resulting molecular orbitals, the bond type is indicated by the nature of the M–O_2_ interaction, and the contributing O–O bond type is indicated in parentheses.

The properties of O_2_ as a ligand are determined by its frontier molecular orbitals (Fig. 1a).^49,52^ As a result of its filled and stable π and σ bonding orbitals, oxygen features a strong O=O double bond and is a poor π donor. Each π* orbital contains an unpaired electron, giving rise to the *S* = 1 ground state of dioxygen. Finally, O_2_ features a high-energy vacant σ*(2p_*z*_) orbital. One- or two-electron reduction results in partial or complete filling of the π* orbitals and the formation of superoxide (O_2_^−^) or peroxide (O_2_^2−^) ligands, respectively. Successive reductions weaken and break the O–O π bond, simultaneously stabilizing the σ*(2p_*z*_) orbital such that it can participate in back-bonding interactions.^53^ While it is generally useful to classify oxygen as a ligand according to the level of reduction, from neutral dioxygen to peroxide, transition metal–O_2_ complexes can be highly covalent, which can complicate the assignment of formal oxidation states.

### 2.2 Dioxygen complexes

Stable metal–O_2_ molecular complexes are rare because neutral O_2_ is generally a poor ligand. Nonetheless, there are cases where O_2_ binding induces a perturbation of the metal electronic structure that imparts stability in the absence of appreciable net electron transfer. For example, the Pauling and Coryell model of hemoglobin involves O_2_ binding to an initially high-spin (*S* = 2) heme iron(ii) center, resulting in singlet O_2_ bound to a low-spin (*S* = 0) iron(ii).^54^ This model is qualitatively correct for the picket fence iron porphyrin, Fe^II^(TpivPP)(1-MeIm) (TpivPP = meso-tetra(α,α,α,α-*o*-pivalamidophenyl)porphyrin; 1-MeIm = 1-methylimidazole),^51,55,56^ a hemoglobin model complex, but is not fully descriptive of hemoglobin (see Section 2.6).^57^ While there is no discrete electron transfer from iron to O_2_ in this complex, the binding is nonetheless very exothermic in the solid state (Δ*H* = −65.3 kJ mol^−1^, Table 3), and importantly reversible.^51^ Indeed, no degradation of the complex was observed over the course of more than 200 O_2_ binding/vacuum cycles. This strong yet reversible binding stems in part from strong backbonding from the metal into the O_2_ π* orbital, enabled by the high degree of porphyrin-iron covalency.^56^ Additionally there is σ-donation from O_2_ to the d_*z*^2^_ orbital of iron. These interactions collectively weaken the O–O bond, resulting in a redshifted O–O stretch of 1159 cm^−1^, which is within the diagnostic range for superoxide.^58^ This example illustrates the limitations of formal assignments in highly covalent metal–O_2_ interactions and demonstrates that the O–O stretching frequency is not always sufficient to assign the degree of O_2_ reduction definitively.^59^ Furthermore, this result suggests targeting iron-based frameworks with highly covalent bonding between metal and linker as a strategy to achieve strong, reversible O_2_ binding.

**Table 3:** O_2_ binding properties of notable biological and synthetic examples

Compound	−Δ*H* (kJ mol^−1^)	−Δ*S* (J mol^−1^ K^−1^)	Δ*G*_298_ (kJ mol^−1^)	M–O_2_ assignment	Ref.
Fe(TpivPP)(1-MeIm)O_2_^a^	65.3	183.0	−10.8	Fe^II^–O_2_	55
Co(TpivPP)(1-MeIm)O_2_^a^	55.6 ± 3.8	192.5 ± 12	1.70	Co^III^–O_2_^−^	62
(Hemoglobin)O_2_^b^	43.1 ± 4.6	74.1 ± 15.5	−21.0	Fe^II^–O_2_/Fe^III^–O_2_^−^	91
(Hemoglobin)(O_2_)_4_^c^	85.0 ± 18.0	190.6 ± 60.2	−28.1	Fe^II^–O_2_/Fe^III^–O_2_^−^	91
[Cu_2_(N4PY2)(O_2_)]^2+^^d^	58 ± 2	165 ± 8	−9.0	Cu_2_O_2_^2−^	64
(Hemocyanin)O_2_^e^	46	67	−26	Cu_2_O_2_^2−^	50

a Collected in the solid state. 1-MeIm = *N*-methylimidazole.

b Binding of the O_2_ to the first site in human hemoglobin in a buffer solution with pH = 7.6.

c Binding of O_2_ to the fourth site human hemoglobin in a buffer solution with pH = 7.6.

d N4 = *N*^1^*N*^1^*N*^4^*N*^4^-Tetrakis(2-(pyridin-4-yl)ethyl)butane-1,4-diamine.

e Binding of O_2_ to P interruptus hemocyanin in buffer solution with pH = 9.6.

### 2.3 Superoxide (O_2_^−^) complexes

Metal–O_2_^−^ complexes form upon transfer of a single electron from a reducing metal center (*e.g.*, Cr^II^, Mn^I^, Fe^II^, Ni^I^, and Cu^I^) into the half-filled π* orbital of O_2_.^52^ Population of this antibonding orbital weakens the π bond of O_2_, resulting in a modest elongation of the O–O distance from 1.21 to 1.29–1.35 Å, as well as a redshift of the O–O stretching mode from 1555 cm^−1^ to 1050–1200 cm^−1^ (Fig. 1b).^23,60^ Superoxide may bind in either a side-on or an end-on mode. The end-on binding mode involves localization of negative charge on the proximal oxygen and a bent M–O–O angle. Side-on binding, while potentially more sterically demanding, can improve metal–superoxide orbital overlap. For both binding modes, the unpaired electron on superoxide resides in a π* orbital, and magnetic coupling between the superoxide and metal center is determined by overlap of this orbital with half-filled metal d orbitals. For example, in an end-on d^9^ copper(ii) superoxide complex (Fig. 1b), the singly occupied d_*x*^2^−*y*^2^_ orbital is orthogonal to the superoxide π* non-bonding orbital, affording an *S* = 1 ground state that arises from ferromagnetic coupling between the metal and O_2_^−^.^61^

The formation of end-on superoxide upon O_2_ binding is also well-documented for five-coordinate cobalt(ii) Schiff base complexes. Oxygen binding in solution results in the formation of a superoxide ion bound to cobalt(iii), with enthalpies ranging from −33 to −77 kJ mol^−1^, and a corresponding high entropic penalty of >150 J mol^−1^ K^−1^.^24^ The oxygenation of cobalt(ii) Schiff base complexes in solution is often reversible, although it is very dependent on solvent and temperature. Particularly at high temperature and in non-polar solvents, the formation of a Co^III^_2_(μ^2^-O_2_^2−^) species can occur, which is usually an irreversible process.^24^ Another well-known Co^III^–O_2_^−^ complex is Co(TpivPP)(1-MeIm)(O_2_), a model for cobalt-substituted hemoglobin that reversibly binds O_2_ (Table 3).^58,62,63^

### 2.4 Peroxide (O_2_^2−^) complexes

In general, peroxide complexes form upon transfer of two electrons from one or more metal centers to fill the formerly half-occupied π* orbitals of O_2_. In compounds featuring a metal prone to two-electron chemistry, such as titanium(ii), manganese(ii), or a second- or third-row transition metal, both electrons may derive from the same metal center.^52^ Reduction of O_2_ to peroxide is associated with significant lengthening of the O–O bond, from 1.21 to 1.40–1.50 Å, as well as a pronounced redshift in the O–O stretching mode to approximately 800–1050 cm^−1^ (Fig. 1c).^60^ In mononuclear complexes, peroxide tends to bind in a side-on fashion, as there is no efficient mechanism for polarizing the O_2_^2−^ charge on a single oxygen atom (Fig. 1c). One notable peroxide complex is oxy-hemocyanin, which features a binuclear copper(ii) core with a side-on bridging O_2_^2−^.^27^ This oxygen-binding protein has inspired the development of a diverse class of molecular mimics,^50^ including [Cu_2_(N4PY2)(O_2_)]^2+^ (N4 = *N*^1^,*N*^1^,*N*^4^,*N*^4^-tetrakis(2-(pyridin-4-yl)ethyl)butane-1,4-diamine), which binds O_2_ reversibly in solution.^64^ Interestingly, increasing temperature and decreasing O_2_ pressure in the headspace of the reaction vessel enables repeated cycling between oxygenated and deoxygenated forms. This molecule binds O_2_*via* single-electron transfer from each copper(i), and this event is associated with an enthalpy of −58 kJ mol^−1^ and an entropy of −165 J mol^−1^ K^−1^, such that the reaction is exergonic at 298 K (Table 3).

### 2.5 Metal and ligand selection and secondary-sphere effects

**Fig. 2:**
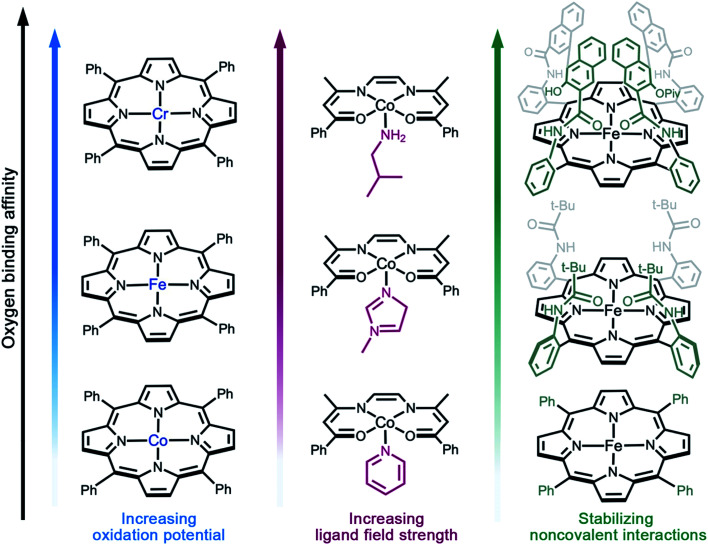
Illustration of design strategies from molecular chemistry to enhance O_2_ binding affinity. (a) Utilizing early- to mid-transition metals,^67–69^ (b) increasing the ligand field strength,^71^ and (c) using bulky side groups that engage in stabilizing non-covalent interactions with the reduced O_2_ species.^55,67,82^ Axial solvent ligands are omitted for clarity; in the single coronet iron porphyrin (top right), the distal two naphthalene molecules have been modified for simplicity.

As discussed above, reversible O_2_ binding at open metal sites can generate a spectrum of O_2_ species and binding modes. Further fine-tuning of O_2_ binding affinity can be accomplished by changing the metal identity in an isostructural series of compounds, perturbing the electronic structure of the metal *via* ligand modifications,^61–63^ or by altering the steric and noncovalent interactions in the secondary coordination sphere (Fig. 2).^33,49,65^ The first effect is well-illustrated by the series of complexes (TPP)M(O_2_)(py) (TPP = tetraphenylporphyrin; M = Cr^II^, Fe^II^, or Co^II^; py = pyridine), wherein the equilibrium constant for oxygenation increases from Co to Cr (Fig. 2a).^66–69^ Indeed, O_2_ binding in Cr(TPP)(py) is irreversible, even at low temperatures.^68^

Examples of electronic structure perturbation by ligand modification abound for divalent first-row metal complexes.^49,65,69–71^ In numerous cobalt(ii) five-coordinate Schiff base and five-coordinate porphyrin complexes, cobalt adopts a low-spin, d^7^ configuration (*S* = 1/2), with the unpaired electron residing in the d_*z*^2^_ orbital.^72–75^ Oxygen binds end-on to the metal site, mediated by a d_*z*^2^_*–*π* orbital interaction that enables electron transfer.^76^ It is possible to destabilize the d_*z*^2^_ energy by changing the axial ligand, which lowers the potential for metal-centered oxidation, thereby increasing O_2_ affinity (Fig. 2b).^49,65,76,77^ In addition, derivatization of the axial ligand can modify the σ and π bonding contributions to oxygen binding. Analogously, subtle modifications to the linker in cobalt(ii) frameworks have been shown to give rise to substantial differences in O_2_ binding enthalpies (see Section 3.5).

Noncovalent interactions can also stabilize bound O_2_ or promote reversible O_2_ binding, as exemplified in several biomimetic systems,^55,78–85^ and hydrogen bonding is known to stabilize the reduced O_2_ species bound in myoglobin and hemoglobin.^70,86^ Additionally, incorporating steric bulk around the metal–O_2_ adduct can stabilize reactive O_2_ intermediates. For example, iron(ii)–porphyrin compounds undergo irreversible oxidation to form oxo-bridged dinuclear species in solution.^87^ In contrast, the picket fence iron(ii) porphyrin Fe^II^(TpivPP)(1-MeIm)_2_ features substantial steric bulk around the metal center, which prevents bridge formation and enables reversible O_2_ binding at 25 °C (Fig. 2c).^55^ More recent strategies combining steric protection with favorable non-covalent interactions have also proven fruitful. For example, single-coronet and twin-coronet iron(ii) porphyrins featuring hydroxyl-functionalized dinaphthalene moieties have been designed to replicate the hydrophobic environment of the active sites in hemoglobin and myoglobin and to promote biomimetic hydrogen bonding interactions with bound O_2_.^82,83^ Despite their ubiquity in biology and small molecule chemistry, secondary sphere interactions remain underutilized in MOF chemistry and represent a worthwhile target for future research in the design of O_2_-selective materials.^88^

### 2.6 Lessons from biology: cooperative O_2_ binding

Hemoglobin is the prototypical iron-containing O_2_ transport protein and features four subunits, with each containing a heme active site.^89,90^ The heme iron(ii) center is a five-coordinate species with the fifth site occupied by an axial histidine ligand, resulting in a high spin (*S* = 2) ground state. Dioxygen binds the first heme site with an enthalpy of −43.1 kJ mol^−1^, yielding a diamagnetic species with an end-on bound O_2_.^91^ A recent study of oxyhemoglobin using X-ray spectroscopy and a valence bond configuration interaction multiplet model revealed mixed low spin Fe^II^/Fe^III^ character with at least 50% iron(iii), reflecting a highly covalent Fe–O_2_ interaction.^57^ The change in the iron(ii) spin state upon O_2_ binding is associated with the depopulation of iron-based orbitals with σ* character, resulting in a contraction of the iron–nitrogen bonds. This local structural change is propagated to the global protein structure *via* the axial histidine, effecting a transition from the so-called ‘tense’ state (low O_2_ affinity) to the ‘relaxed’ state (high O_2_ affinity).^89^ This change facilitates subsequent O_2_ binding events at the other heme sites. For example, O_2_ binding to the fourth heme is associated with an enthalpy of −85.0 kJ mol^−1^ (Table 3).^91^ This cooperativity is key to the function of hemoglobin, enabling the capture and release of relatively large quantities of O_2_ in response to a small change in oxygen partial pressure, and is associated with a sigmoidal O_2_ binding curve. An unrealized goal in the design of O_2_-selective MOFs is to harness cooperativity to enhance material working capacities for O_2_ separations, an approach that has proven to be effective for other gaseous adsorbents, including CO_2_ and CO.^92,93^ As discussed below (see Section 3.4), a triazolate-based framework featuring high-spin iron(ii) has been shown to react with O_2_ to form low-spin iron(iii) bound to superoxide, reminiscent of the mechanism of O_2_ binding in hemoglobin.^45^ As such, further exploration of the reduction of O_2_ to superoxide by iron-containing MOFs of different topologies may be a fruitful direction in pursuit of cooperative O_2_ binding in MOFs.

## 3. Oxygen-selective metal–organic frameworks

### 3.1 Enthalpy and entropy considerations for O_2_ adsorbents

The enthalpy of O_2_ binding at an open metal site is a critical parameter for evaluating the adsorption performance of a material. Indeed, the energy consumption of an adsorption process depends heavily on the enthalpy of adsorption, and in some cases, the enthalpy can represent half of the total energy required for adsorbent regeneration.^94^ As such, the O_2_ binding enthalpy is a critical metric to optimize in the design of a commercially relevant O_2_-selective MOF, and one that can be judiciously tuned using synthetic chemistry. Perhaps unsurprisingly, there has been a historical focus on this particular parameter. In general, the lower the O_2_ binding enthalpy of a material, the lower the corresponding energy demand for adsorbent regeneration. However, if the enthalpy of O_2_ binding is too low, the material may not exhibit sufficient selectivity for O_2_ over N_2_.

The enthalpy of N_2_ binding at open metal sites has been experimentally determined for a number of frameworks^44,45,95,96^ with corresponding values typically ranging from −10 to −25 kJ mol^−1^ (*vs.* Δ*H* = −22.5 kJ mol^−1^ for Li-LSX^97^). Accordingly, candidate MOFs for O_2_-selective air separations should ideally exhibit O_2_ binding enthalpies well above this range. However, materials that exhibit O_2_ binding enthalpies that surpass −80 kJ mol^−1^ may not afford any energy savings over the current industrial benchmark, N_2_-selective Li-LSX, due to the energy required to regenerate the O_2_ adsorbent (see ESI Section S2†). Thus, to provide a baseline for analysis, we propose −45 kJ mol^−1^ as a reasonable target O_2_ binding enthalpy, which would correspond to a material regeneration energy that is ∼50% less than that required for Li-LSX.^28,97^ While an even higher enthalpy of adsorption is likely to be associated with greater O_2_/N_2_ selectivity, it would come at the cost of greater energy demand for regeneration.

While the enthalpy of O_2_ binding is a key factor that will impact the overall energy of adsorbent-based air separations, this parameter must be considered together with the entropy of adsorption. Indeed, it is Δ*G*, and not Δ*H* alone, that determines the adsorption properties of a material. In particular, Δ*G* for the primary O_2_ binding site will determine the working capacity under a given set of conditions.^98^ This information is necessary to establish the potential performance of the material in an adsorptive process (see Section 3.5). As such, a binding enthalpy of −45 kJ mol^−1^ is desirable as long as the corresponding entropy of binding results in an appropriate Δ*G* for the adsorption process. Relative to binding enthalpy, it is much more difficult to tune the entropy of O_2_ binding *via* metal and/or linker modifications. Entropies of adsorption are not typically reported in the literature, and currently there is no consistent method for reporting both Δ*H* and Δ*S* for O_2_-selective adsorbents.^99^ As such, to enable a more rigorous comparison of materials in this Perspective, we calculated enthalpy and entropy values for several key O_2_-selective frameworks using published Langmuir–Freundlich fits or O_2_ adsorption data (see ESI Sections S4 and S5 for details and Tables S1–S9†).^44–47,88^ More generally, we suggest that routine determination of binding enthalpies and entropies is a critical facet of materials characterization, requiring minimal additional work, that will enable more rigorous development and benchmarking of MOFs for practical application.

### 3.2 Key framework structure types

**Fig. 3:**
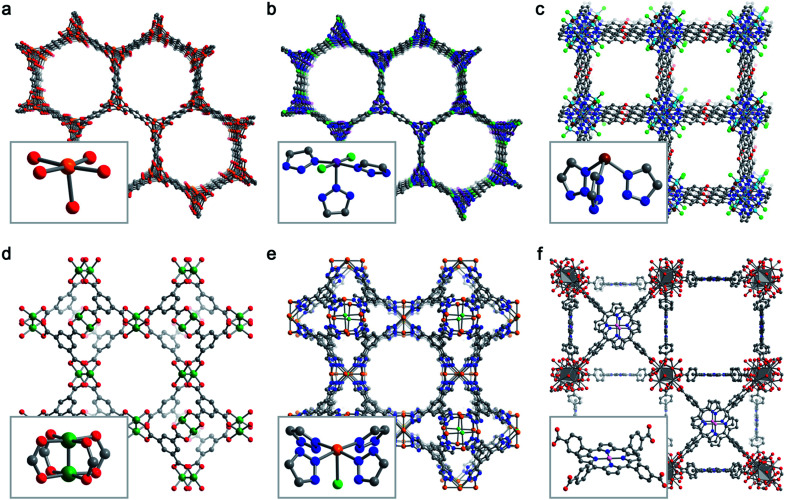
Representative structures obtained from powder X-ray diffraction (a, b, e), powder neutron diffraction (c, d), or single-crystal X-ray diffraction (f) depicting the six MOF structure types discussed in Section 3.2. Insets show the first coordination sphere of each open metal site. (a) Fe_2_(dobdc),^43^ (b) Co_2_Cl_2_(bbta),^88^ (c) Cu^I^-MFU-4*l*,^110^ (d) Cu_3_(btc)_2_,^115^ (e) Fe-BTTri,^124^ and (f) Mn-PCN-224.^47^ Orange, purple, green, pink, grey, red, and blue spheres represent Fe, Co, Cu, Mn, C, O, and N atoms, respectively; H atoms are omitted for clarity.

Despite the effectively limitless structural variability possible for metal–organic frameworks, a relatively limited number of frameworks have been studied for selective O_2_ adsorption. Here, we briefly describe six key framework structure types that have been studied for selective O_2_ capture (Fig. 3).

#### 3.2.1 M_2_(dobdc)

The M_2_(dobdc) (M = Mg^II^, Mn^II^, Fe^II^, Co^II^, Ni^II^, Cu^II^, Zn^II^; dobdc^4−^ = 2,5-dioxido-1,4-benzenedicarboxylate; MOF-74, CPO-27)^95,100–103^ family of frameworks features one-dimensional hexagonal pores with vertices lined by helical chains of coordinatively unsaturated divalent metal centers (Fig. 3a). The metals exhibit a square pyramidal coordination geometry, with open coordination sites facing into the pore interior, and are ligated by weak-field carboxylate and aryloxide ligands. These frameworks are particularly attractive, owing to their facile syntheses, tunability, and high density of open metal sites (6.54 mmol g^−1^ in the case of Fe_2_(dobdc), Table 4). Oxygen adsorption has been studied in the Mn^2+^, Fe^2+^, Co^2+^, Ni^2+^ and Cu^2+^ variants of M_2_(dobdc).^43,95,96^

**Table 4:** Theoretical and experimental O_2_ capacities, IAST O_2_/N_2_ selectivities, reported binding enthalpies, and reported O–O infrared stretching frequencies for selected metal–organic frameworks, organized from highest to lowest binding enthalpy

MOF	Theoretical O_2_ capacity^a^		Experimental uptake at 1 bar^b^			IAST/T (K)	−Δ*H*_O_2__ (kJ mol^−1^)	O–O^c^ (cm^−1^)	Ref.
	mmol g^−1^	wt (%)	mmol g^−1^	wt%	*T* (K)				
Cr-BTT	3.94	11.2	2.57	7.59	298	2570/298	65	1193	123
Cr_3_(btc)_2_	6.43	17.1	3.9	11	298			1129	42
Cu^I^-MFU-4*l*	1.69	5.13	2.33	6.94	233		53		46
Fe-BTTri	3.91	11.1	5.90	15.9	195	27/195	51	1199	45
Mn-PCN-224	0.73	2.3	0.86	2.7	298		49.6(8)	984	47
Co_2_(OH)_2_(bbta)	6.45	17.1	7.57	19.5	195	42/195	49(2)	1151	88
Co-BDTriP	3.87	11.0	4.8	13	195	105/195	47(1)		44
Fe_2_(dobdc)	6.54	17.3	8.16	20.7	211	8/214	41	1129	43
Co-BTTri	3.86	11.0	4.8	13	195	41/195	34(1)		44
Fe-PCN-224	0.73	2.3	0.26	0.83	298		34(4)		129
Co-PCN-224	0.73	2.3	2.00	6.02	195		15.2(6)		130

a Calculated based on gravimetric density of open metal sites.

b For Cr-BTT, Cr_3_(btc)_2_, and Fe_2_(dobdc), reported uptakes in wt% were converted to mmol g^−1^; all other framework capacities were reported in mmol g^−1^ and converted to wt%.

c Stretching frequencies reported at temperatures associated with reversible O_2_ binding.

#### 3.2.2 M_2_X_2_(bbta)

Analogous to M_2_(dobdc), the M_2_X_2_(bbta) (M = Mn^II^, Fe^II^, Co^II^, Ni^II^; X = Cl^−^, Br^−^, F^−^, OH^−^; H_2_bbta = 1*H*,5*H*-benzo(1,2-*d*:4,5-*d*′)bistriazole) structure type^88,93,104–107^ also features one-dimensional hexagonal channels with pore vertices lined by coordinatively-unsaturated, square pyramidal M^II^ sites. However, the primary metal coordination sphere is occupied by two basal and one apical triazolate ligands and two μ^2^-halide or hydroxide ligands (Fig. 3b). Several expanded-pore analogues of this family have also been developed—including the recently reported vanadium variant^41^—of the type M_2_Cl_2_(btdd) (H_2_btdd = bis(1*H*-1,2,3-triazolo[4,5-*b*],[4′,5′-*i*])dibenzo[1,4]dioxin).^105,108^ While only Co_2_X_2_(bbta) (X = Cl^−^ and OH^−^) has been studied for O_2_ separations,^88^ the tunability and metal site density of this material class render it a promising target for further exploration. As an example, the metal site density of Co_2_(OH)_2_(bbta) is 6.45 mmol g^−1^, comparable to the M_2_(dobdc) series (Table 4).

#### 3.2.3 M-MFU-4*l*

The framework MFU-4*l*, or Zn_5_Cl_4_(btdd)_3_, features pentanuclear zinc nodes, each bridged by btdd^2−^ linkers to six other nodes, resulting in three-dimensional pore system with square pore openings.^109^ An octahedral zinc(ii) ion sits at the center of each pentanuclear node, and four tetrahedral zinc(ii) sites capped by terminal chloride ligands occupy the periphery. Approximately two of these four tetrahedral zinc sites can be post-synthetically exchanged with other divalent metals to yield M^II^-MUF-4*l* (M = Mn, Fe, Co, Ni, and Cu).^110^ Reduction of Cu^II^-MFU-4*l* yields Cu^I^-MFU-4*l*, which features trigonal pyramidal copper(i) sites (Fig. 3c).^46^ Although their density is low (1.69 mmol g^−1^), these open copper(i) sites interact strongly (Δ*H* = −52.6(6) kJ mol^−1^) and reversibly with O_2_. Interestingly, a recent investigation of O_2_ binding in Cu^I^-MFU-4*l* using near-edge X-ray absorption fine structure spectroscopy and DFT revealed that the interaction between copper(i) and O_2_ is highly covalent, and that O_2_ most likely binds side on at the metal site in its triplet configuration.^111^ Given the high tunability^110,112,113^ of this structure type, it could serve as an excellent platform to optimize O_2_ binding through ligand modification, as an example. We note that the isoreticular framework MFU-4, or Zn_5_Cl_4_(bbta)_3_, is known,^114^ and although it has not been demonstrated, if the peripheral Zn^II^–Cl units in the structure can be replaced with Cu^I^, then the corresponding material could exhibit a significantly greater O_2_ capacity than Cu^I^-MFU-4*l*.

#### 3.2.4 M_3_(btc)_2_

As discussed above, the first MOF investigated for O_2_ adsorption was Cr_3_(btc)_2_,^42^ which belongs to a larger family of M_3_(btc)_2_ materials (also known as M-HKUST-1; M = Cr^II^, Fe^II^, Ni^II^, Cu^II^, Zn^II^, Mo^II^, Ru^II^).^115–122^ This cubic framework structure features dinuclear paddlewheel-type nodes, each coordinated by four carboxylate groups provided by the triangular trimesate linkers. Each exposed metal site from each paddlewheel node points into a cavity and is accessible to guest molecules (Fig. 3d). This structure class also features a high density of open metal sites (6.43 mmol g^−1^ for Cr_3_(btc)_2_, Table 4). To date, Cr_3_(btc)_2_ is the only material studied for selective O_2_ uptake in this family. However, solvated Fe_3_(btc)_2_ has been isolated and structurally characterized,^122^ and the permanently porous and fully desolvated framework represents an interesting candidate for further study.

#### 3.2.5 M–Benzenetrisazolates

The frameworks Cr_3_[(Cr_4_Cl)_3_(BTT)_8_]_2_ (Cr-BTT; H_3_BTT = 1,3,5-tri(1*H*-tetrazol-5-yl)benzene),^123^ M_3_[(M_4_Cl)_3_(BTTri)_8_]_2_ (M-BTTri; M = Fe^II^, Co^II^; H_3_BTTri = 1,3,5-tri(1*H*-1,2,3-triazol-5-yl)benzene),^44,45,124^ and Co_3_[(Co_4_Cl)_3_(BDTriP)_8_]_2_ (Co-BDTriP; H_3_BDTriP = 5,5′-(5-(1*H*-pyrazol-4-yl)-1,3-phenylene)bis(1*H*-1,2,3-triazole))^44^ are O_2_ adsorbents belonging to a large family of cubic M–benzenetrisazolate MOFs (Fig. 3e) with open metal site densities close to 4 mmol g^−1^ (Table 4).^125–127^ These sodalite-type structures are built up of truncated octahedra with square nodes formed by four M^2+^ centers bridged by a central μ^4^-Cl and triangular faces formed by benzenetrisazolate linkers. These frameworks are anionic and are charge-balanced by three extra-framework M^II^ ions per formula unit. Each square pyramidal metal center is coordinated by four basal azolate nitrogen atoms and one apical chloride. The material Co-BDTriP is unique in that it features a distribution of cobalt(ii) sites bound by a combination of pyrazolates and triazolates.^44^ Notably, Cr-BTT exhibits one of the highest O_2_ capacities of any framework studied to date, 7.6 wt% at 298 K and 1 bar.^123^

#### 3.2.6 M-PCN-224

The M-PCN-224 frameworks (M = Mn^II^, Fe^II^, Co^II^, Ni^II^, Cu^II^) consist of zirconium cluster nodes connected by tetratopic porphyrin linkers in a cubic architecture.^47,128–132^ The composition of these frameworks is highly variable depending on the synthetic conditions used, and as such it is not straightforward to specify one representative chemical formula. However, a recent study demonstrated that different post-synthetic treatments of the as-synthesized precursor PCN-224 (ref. 133) yield well-defined structures (H_2_tcpp)_3_[Zr_6_O_4_(OH)_4_(CH_3_CO_2_)_6_]_2_ or (H_2_tcpp)_3_[Zr_6_O_4_(μ-OH)_4_(OH)_6_]_2_ (H_2_tcpp = 5,10,15,20-tetrakis(4-carboxyphenyl)porphyrin). For simplicity, in the subsequent discussion and calculations, we assume a formula of M_3_(tcpp)_3_[Zr_6_O_4_(μ-OH)_4_(OH)_6_]_2_ for the M-PCN-224 frameworks. Of the frameworks studied to date, the Mn^II^, Fe^II^, and Co^II^ variants have been shown to selectively adsorb O_2_. The isolated four-coordinate square planar metal centers in M-PCN-224 (Fig. 3f) mimic metalloporphyrin active sites in proteins, including hemoglobin and myoglobin. As the framework structural rigidity precludes binuclear decomposition pathways encountered with molecular compounds, these frameworks serve as important model systems for O_2_ binding in biological systems. However, these MOFs exhibit the lowest metal site densities among the materials considered here (approximately 0.73 mmol g^−1^).

### 3.3 Tuning O_2_ binding through ligand modification

Typically, MOFs that feature open metal sites are constructed using weak-field linkers, such as 2,5-dioxido-1,4-benzenedicarboxylate.^100,133^ As such, the metal nodes are best described as Lewis acidic^134^ and generally interact weakly with oxygen.^95^ A telling example is Co_2_(dobdc), which remains high-spin when bound to CO, and exhibits an O_2_ binding enthalpy of just −18.56(3) kJ mol^−1^.^96^ As discussed in Section 2, strong oxygen binding in molecular cobalt complexes is well documented,^49^ but it was only in 2016 that a cobalt(ii)-based framework with a stronger ligand field than Co_2_(dobdc) was reported. The material Co-BTTri exhibits a modest O_2_ adsorption enthalpy of −34(1) kJ mol^−1^ (Table 4) for loadings up to ∼2 mmol g^−1^ (6 wt%).^44^ Vibrational modes corresponding to the O_2_ adduct could not be observed at room temperature, but calculations suggest there is only partial electron transfer from cobalt(ii) to O_2_ (0.31 electron equivalents). The theoretical uptake of the material, assuming one bound O_2_ at each low-spin cobalt(ii) site, is 3.86 mmol g^−1^ (11.0 wt%); however, the experimental uptake at the cobalt sites was approximately 2.8 mmol g^−1^ (8.2 wt%), as determined from the inflection point in the plot of the isosteric heat of adsorption as a function of loading. This lower than expected capacity is common in frameworks with open metal sites and may be due to the presence of residual coordinated synthesis solvent or blocked pore access^135^ or potentially even metal site vacancies.^136,137^ Interestingly, the N_2_ enthalpy of adsorption in Co-BTTri is only −12(1) kJ mol^−1^,^44^ and this relatively weak affinity for N_2_ suggests that the low-spin cobalt(ii) sites are neither strongly Lewis acidic or π-basic.

Replacement of the triangular BTTri^3−^ linker with the more basic BDTriP^3−^, which features one pyrazolate and two triazolate donors, yields the framework Co-BDTriP.^44^ Characterization of this material *via* single-crystal X-ray diffraction supports the presence of a statistical distribution of five unique cobalt centers coordinated by a combination of triazolates and pyrazolates. Notably, at very low loadings, the isosteric heat of O_2_ adsorption in Co-BDTriP is −47(1) kJ mol^−1^, corresponding to O_2_ bound at approximately 12% of the cobalt(ii) sites in the material. This enthalpy is much larger than the highest binding enthalpy characterized for Co-BTTri (and within the range observed for cobalt(ii) Schiff complexes, see Section 2.3), and was ascribed to O_2_ binding at cobalt sites ligated by three or more pyrazolates. Relative to the enthalpy of O_2_ binding in Co-BTTri at low-loading, this larger value can be rationalized as arising from a greater degree of charge transfer from cobalt(ii) to O_2_, resulting from the more basic (electron-donating) pyrazolate groups. With increasing gas loading, the enthalpy of O_2_ binding in Co-BDTriP decreases, and at a loading of approximately 1 mmol g^−1^, Co-BDTriP and Co-BTTri exhibit similar binding enthalpies. Interestingly, increasing the linker basicity from Co-BTTri to Co-BDTriP has little effect on the enthalpy of N_2_ adsorption. The more basic linker environment in Co-BDTriP may destabilize the d_*z*_^2^ orbital of cobalt(ii) relative to that in Co-BTTri, yielding a less Lewis-acidic metal center and thereby diminishing the σ interaction between the HOMO of N_2_ and cobalt, compensating for any enhanced π-backbonding. Considering the enhancement in O_2_ binding enthalpy at low loadings upon moving from Co-BTTri to Co-BDTriP, a promising target may be the material Co_3_[(Co_4_X)_3_(BTP)_8_]_2_ (hereafter, *Co-BTP*, H_3_BTP = 1,3,5-tri(1*H*-pyrazol-4-yl)benzene), featuring all pyrazolate donors. Such a material isostructural to Co-BTTri and Co-BDTriP has not yet been isolated, although the framework Co_3_(BTP)_2_ has previously been synthesized,^138^ suggesting that appropriate synthetic conditions may yield *Co-BTP*. In general, the results for Co-BTTri and Co-BDTriP indicate that further investigation of suitable frameworks featuring basic azolate-based linkers and cobalt(ii) centers is a worthwhile pursuit.

The open metal sites in Co_2_Cl_2_(bbta) exhibit a square pyramidal metal coordination geometry, similar to that in Co-BTTri and Co-BDTriP. However, the metal sites are ligated by two *trans* basal chloride ions and three triazolates (Fig. 3b).^105^ The O_2_ binding enthalpy of −15 kJ mol^−1^ in Co_2_Cl_2_(bbta)^88^ is significantly lower than in Co-BTTri and Co-BDTriP, and perhaps surprisingly, even lower than in Co_2_(dobdc). Here, the replacement of a strong σ-donating nitrogen ligand with a weakly σ-donating chloride likely attenuates metal-to-oxygen charge transfer, and thus the cobalt(ii) sites in Co_2_Cl_2_(bbta) are both insufficiently reducing and poor Lewis acids.

Replacing the bridging chlorides in Co_2_Cl_2_(bbta) with more basic hydroxides yields the material Co_2_(OH)_2_(bbta).^106^ This framework exhibits an O_2_ binding enthalpy of −49(2) kJ mol^−1^ at low loadings, the highest value reported to date for a cobalt framework.^88^*In situ* variable-temperature diffuse reflectance Fourier transform spectroscopy (DRIFTS) was used to characterize the O_2_ binding and revealed an O–O stretch at 1151 cm^−1^, consistent with a superoxide moiety bound end-on to cobalt(iii). DRIFTS data also indicate that the superoxide is stabilized by hydrogen bonding interactions with the bridging hydroxo groups of the framework, reminiscent of the stabilization of superoxide in oxygen binding proteins. Calculations suggest that this secondary coordination lowers the binding energy of O_2_ by 20 kJ mol^−1^, highlighting the significant impact of subtle coordination sphere changes in isostructural MOFs. This additional interaction also contributes to a very large entropy of adsorption of −186(7) J mol^−1^ K^−1^.

Interestingly, the calculated saturation capacity of the strong O_2_ binding site in Co_2_(OH)_2_(bbta)—determined from fits using a dual-site Langmuir model—was found to be 2.46 mmol g^−1^, only 38% of the theoretical capacity based on open metal site density. However, data from powder X-ray diffraction analysis revealed that the actual occupancy is much higher (approximately 75%), indicating that the chosen adsorption model was not adequate for describing the O_2_ uptake in this material. Together with *in situ* DRIFTS data obtained at different O_2_ loadings, these results revealed that O_2_ binding weakens as a function of loading, a rare example of example of negatively cooperative gas binding in a metal–organic framework.^139^ Notably, the same extended lattice interactions that promote initial strong O_2_ binding contribute to this unusual effect. Indeed, with increasing O_2_ loading, the proportion of cobalt(iii)–O_2_^−^ moieties also increases, rendering neighboring cobalt(ii) sites less electron-donating and therefore less likely to bind O_2_*via* electron transfer. This unexpected result highlights that the nature of electronic communication between metal sites in MOFs must be tuned carefully to achieve desired O_2_ binding properties. Here, the appropriate choice of a secondary metal could potentially give rise to an ordered mixed-metal framework of the type CoM(OH)_2_(bbta) that exhibits a high enthalpy of O_2_ binding in the absence of negative cooperativity. In this case, it may be possible to achieve an O_2_ uptake as high as 3 mmol g^−1^. However, due to the presence of hydrogen bonding interactions, even in this case, O_2_ binding is still likely to be associated with a high entropic penalty. As an alternative, replacing the hydroxide moieties with bridging methoxide or methylthiolate anions would remove such interactions and potentially still provide sufficient reducing power at the metal center to ensure a high binding enthalpy.

### 3.4 Tuning O_2_ binding through metal selection

One of the most well-studied isostructural framework series is M_2_(dobdc) (M = Mn^II^, Fe^II^, Co^II^, Ni^II^, Cu^II^, Zn^II^).^133^ Excluding the iron variant, the open metal sites in this family behave like Lewis acids, and generally, they preferentially bind N_2_ over O_2_,^43,95,96^ given the greater polarizability and quadrupole moment of N_2_ (Table 1).^97^ Characterization of O_2_ binding in the Mn, Co, Ni, and Cu variants revealed that the metal–O_2_ interactions are also predominately electrostatic in nature.^95,96^

The case of O_2_ binding in Fe_2_(dobdc) is unique and can be partly rationalized by the fact that iron(ii) exhibits a lower ionization energy than Mn^II^, Co^II^, Ni^II^, and Cu^II^. Uptake of O_2_ in Fe_2_(dobdc) results in very steep adsorption isotherm and occurs *via* superoxide or peroxide formation, depending on the temperature.^43^ At 211 K, ∼90% of the iron sites reversibly bind O_2_, with a total uptake for the material of 6.54 mmol g^−1^ (17.3 wt%) at 1 bar. Notably, the material is stable to repeated cycling and exhibits no loss in capacity at 211 K over the course of at least 13 adsorption/desorption cycles. The enthalpy of O_2_ adsorption in this material was calculated to be −41 kJ mol^−1^. Although this value is lower than the binding enthalpy determined for Co_2_(OH)_2_(bbta), the associated O_2_ moiety in Fe_2_(dobdc) is more reduced than that bound in Co_2_(OH)_2_(bbta), as judged from their respective O–O stretches of 1129 and 1151 cm^−1^ (Table 4). The enhanced stabilization of bound O_2_ in Co_2_(OH)_2_(bbta) relative to Fe_2_(dobdc) can again be ascribed to the substantial enthalpic contribution from hydrogen bonding (as discussed above). At room temperature, O_2_ binding in Fe_2_(dobdc) results in irreversible formation of iron(iii)–peroxide species at half of the iron sites, with the second reducing equivalent provided by the remaining iron sites. As expected, the O–O bond of the reduced moiety is substantially weakened, and a very low O–O stretch of 790 cm^−1^ was characterized *via* IR spectroscopy. The ordered substitution of 50% or more of the iron(ii) sites in Fe_2_(dobdc) with another divalent metal could potentially suppress the electron transfer between metal sites that leads to irreversible peroxide formation and enable access to a material exhibiting high, reversible O_2_ uptake. Of note, while metal⋯metal communication in Fe_2_(dobdc) has a deleterious effect on O_2_ uptake, it is interesting to consider how the participation of two adjacent metal sites in one reduction and binding event, as observed here, could be manipulated *via* linker and secondary-sphere interactions to achieve positive cooperativity in O_2_ binding.

The framework Fe-BTTri was recently reported, enabling preliminary evaluation of the influence of metal identity on O_2_ binding in M-BTTri. Interestingly, the local iron coordination environment in this material bears some resemblance to the heme site of hemoglobin.^45^ At 195 K, 64% of the exposed high-spin iron(ii) framework sites bind O_2_*via* superoxide formation with an enthalpy of −51 kJ mol^−1^, which is 1.5 times that of the binding enthalpy in Co-BTTri. The material reversibly adsorbs 3.3 mmol g^−1^ (9.6 wt%) of O_2_ at 195 K and 210 mbar over the course of 5 cycles. *In situ* DRIFTS data obtained at 195 K revealed an O–O stretch at 1199 cm^−1^, which, together with Mössbauer data, confirms the presence of superoxide bound to low-spin iron(iii), reminiscent of the mechanism of O_2_ binding in hemoglobin (Section 2.6). This complete electron transfer from iron(ii) to O_2_ contrasts with the partial electron transfer upon O_2_ binding in Co-BTTri. Above 258 K, O_2_ binding is irreversible.

The M-PCN-224 (M = Mn^II^, Fe^II^, Co^II^) framework class is another instructive example to evaluate the importance of metal identity for O_2_ binding in MOFs.^47,129,130^ In contrast to what is observed for M_2_(dobdc), the enthalpy of O_2_ binding in M-PCN-224 increases from cobalt to iron to manganese, consistent with the general trend observed for metalloporphyrin complexes (Section 2). Note, however, that O_2_ binding to Mn-PCN-224 (ref. 47) is a two-electron process, whereas one-electron reduction occurs upon O_2_ uptake in the other two frameworks. In Mn-PCN-224, 85% of the manganese(ii) sites bind O_2_ with an enthalpy of −49.6(8) kJ mol^−1^. The resulting adduct is an η^2^-peroxomanganese(iv), and the reaction is reversible upon purging with argon.

The framework Fe-PCN-224 (ref. 129) provides a rare example of a base-free heme model. In this material, the high-spin ferrous centers bind O_2_ at 195 K to form low-spin iron(iii)–superoxo moieties, as characterized using single-crystal X-ray diffraction and Mössbauer spectroscopy data collected at 100 K. Oxygen binding is only appreciable at 195 K and below, and from low-temperature (141, 156, and 195 K) isotherm data the O_2_ binding enthalpy was determined to be −34(4) kJ mol^−1^, consistent with the formation of a superoxo moiety. Notably, this value is substantially lower than that associated with O_2_ binding in hemoglobin as well as in biomimetic compounds that feature a bound axial imidazole.^51,91^ Using reported isotherm data obtained at higher temperatures (226, 273, and 298 K), we calculated an even lower binding enthalpy of −19(2) kJ mol^−1^. The large difference in the enthalpy values for the given temperature regimes suggests that binding of O_2_ at and near room temperature is associated with the formation of an iron–O_2_ adduct distinct from that observed at lower temperatures. In all, these data suggest that electron transfer from iron to O_2_ in this system is temperature-dependent, and further studies are warranted to investigate this possibility.

Dioxygen binding in Co-PCN-224 (ref. 130) results in the formation of a low-spin Co^III^–superoxide complex, as characterized *via* single-crystal X-ray diffraction and EPR spectroscopy performed at 85 K. Oxygen adsorption data collected at higher temperatures (113, 141, 156, and 195 K) were used to calculate an enthalpy of O_2_ binding of −15.2(6) kJ mol^−1^, which is much lower than that associated with superoxide formation in Co(TpivPP)(1-MeIm) (Table 3). It is possible that at the higher temperatures associated with isotherm data collection, O_2_ binding results in a Co^II^–O_2_ species. Indeed, an enthalpy of −15.2(6) kJ mol^−1^ is more consistent with reported enthalpies of −15 and −18.56(3) kJ mol^−1^ associated with the formation of Co^II^–O_2_ adducts in Co_2_Cl_2_(bbta)^88^ and Co_2_(dobdc),^96^ respectively. In all, the above results suggest that the detailed investigation of O_2_ binding modes in Fe-PCN-224 and Co-PCN-224 as a function of temperature, including the determination of enthalpies and entropies, represents a worthwhile fundamental study.

### 3.5 Evaluating optimal Δ*G* for O_2_-selective adsorptive processes

As discussed above, values of Δ*H* are traditionally reported for O_2_-selective adsorbents, but the Δ*G* of O_2_ binding is a more meaningful metric for evaluating the performance of a material under practical working conditions. In particular, the Δ*G* of O_2_ binding is a key parameter for determining the useable surface coverage, Δ*θ*, defined as the difference between the surface coverage under adsorption (*θ*_ads_) and desorption (*θ*_des_) conditions (see ESI Section S6†).^98^ The maximal value of Δ*θ* under a given set of working conditions is 1, corresponding to complete coverage of the primary O_2_ binding sites upon adsorption (*θ*_ads_ = 1) and zero coverage upon desorption (*θ*_des_ = 0). Thus, for a given set of target conditions, it is possible to calculate an optimal Δ*G* value that will maximize theoretical useable capacity. Note that this analysis is intended as a useful starting point, and a comprehensive evaluation of a candidate material will necessarily consider other factors such as O_2_/N_2_ selectivity (see Section 3.6) and adsorption kinetics.

The specific working conditions for an adsorptive air separation process depend on a multitude of factors, including capital and operating costs, production scale, location, and load variability.^12,18,94,140,141^ For the purposes of the present assessment, we considered a VSA process under two sets of working conditions, namely adsorption of air at 1 bar and 298 K and desorption at 10 or 1 mbar, which are common minimum pressures for dry reciprocating pumps. Based on progress achieved thus far in the development of O_2_-selective MOFs and the inherent tunability of these materials, we propose that these conditions represent reasonable targets and additionally would afford considerable costs and energy savings relative to current technologies.

**Fig. 4:**
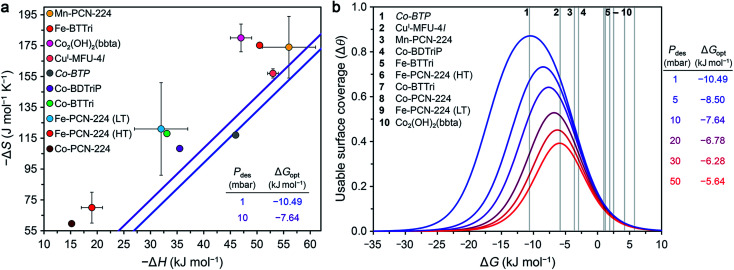
(a) Calculated enthalpy and entropy values of O_2_ binding in nine selected frameworks as discussed in the text (colored circles). The optimal Gibbs free energies (Δ*G*_opt_) that maximize O_2_ useable surface coverage (Δ*θ*) at 298 K for a VSA process with adsorption at 0.21 bar and desorption 1 and 10 mbar are plotted as blue and purple lines, respectively (see Section S6 of the ESI†). (b) O_2_ useable surface coverage at 298 K plotted as a function of Δ*G* for a range of desorption pressures, following adsorption at 0.21 bar. The optimal Gibbs free energy (Δ*G*_opt_) associated with the maximum useable capacity at each pressure is also given. The Gibbs free energies of O_2_ binding for the same frameworks in (a) are shown in the plot as grey bars. As a result of the non-linear relationship between Δ*θ* and Δ*G*, minor deviations from Δ*G*_opt_ have a significant impact on the value of Δ*θ*. See Tables S13 and S14† for the calculated working capacity of each framework under the considered VSA process conditions.

Optimal surface coverage under these two sets of conditions is achieved for Δ*G*_298_ values of −10.49 and −7.64 kJ mol^−1^, for desorption at 1 and 10 mbar, respectively. These values are shown as blue and purple lines, respectively, in the plot of Δ*S vs.* Δ*H* given in Fig. 4a. The points plotted in Fig. 4a represent the calculated values of Δ*G* at 298 K associated with O_2_ binding at the open metal sites in eight reported frameworks and the hypothetical material *Co-BTP*, based on calculated Δ*S* and Δ*H* values (see the ESI Section S5 for details and Table S11†). An implicit assumption in comparing these data is that the Δ*H* and Δ*S* values for each framework are temperature-independent, given the range of temperatures (typically well below 298 K) used to collect adsorption data. While this assumption is not always valid, as discussed previously for Fe- and Co*-*PCN-224, these results are intended to serve as a proof-of-concept demonstration of using Δ*G* as a key parameter for more comprehensive benchmarking than has been accessible based on Δ*H* alone. Overall, the data in Fig. 4a highlight that O_2_ binding in most of the MOFs analyzed is not sufficiently exergonic for achieving optimal useable surface coverage under the considered conditions. The one outlier is Cu^I^-MFU-4*l*, for which the Δ*G* of O_2_ binding at the open copper(i) sites is calculated to be −6(1) kJ mol^−1^. The hypothetical material *Co-BTP* is also predicted to have a near-optimal Δ*G* of −11 kJ mol^−1^. Interestingly, recall the Δ*G*_298_ of O_2_ binding in the molecule Fe(TpivPP)(1-MeIm)_2_ is −10.8 kJ mol^−1^ (Table 3), which suggests that the pursuit of new framework types featuring analogous iron–porphyrin units may be a promising design strategy. Ultimately, using these values as a guideline for evaluating materials must be done with caution, considering that an optimal Δ*G*_298_ value must be associated with a sufficiently high enthalpy of adsorption to ensure selectivity over N_2_ while minimizing regeneration energy.

We also determined Δ*θ* as a function of Δ*G* for several desorption pressures ranging from 1 to 50 mbar, as shown in Fig. 4b, where the same frameworks from Fig. 4a are indicated as numbered grey lines. As a result of the non-linear dependence of Δ*θ* on Δ*G* and the desorption pressure (see ESI Section S6†), minor deviations in Δ*G* and *P*_des_ can dramatically impact Δ*θ*. For example, the lowest desorption pressures are associated with the largest gains in surface coverage. Only Cu^I^-MFU-4*l* and *Co-BTP* exhibit Δ*G* values that give rise to optimal useable surface coverage. Targeting materials that exhibit similar open metal sites and associated enthalpy and entropy values is a worthwhile pursuit.

The combined use of high temperature and vacuum to regenerate an adsorbent, in a so-called vacuum temperature swing adsorption (VTSA) process, can give rise to higher useable surface coverages than accessible with the use of VSA or TSA alone.^14^ Additionally, incorporating a temperature swing for desorption enables the recovery of waste heat, albeit generally at the expense of slower cycle times. We assessed the performance of the same MOFs considered above in a VTSA process involving adsorption of air at 298 K and 1 bar (0.21 mbar O_2_) and desorption at 338 K and 10 mbar (Table 5). Under these conditions, *Co-BTP* and Cu^I^-MFU-4*l* again exhibit the highest useable surface coverages of all the frameworks, with Δ*θ* = 0.86 and 0.71, respectively. These values are comparable to that achieved using a VSA process and desorption at 1 mbar and 298 K. In other words, the Δ40 K temperature swing compensates for the 10-fold weaker vacuum in this process. Finally, we emphasize that the foregoing analysis is helpful only to compare the Δ*G* of adsorption across different frameworks and relative to a defined process condition, and this parameter alone is not sufficient to qualify benchmark adsorbents. It is also critical to consider other factors, such as the density of binding sites in an adsorbent, as well as its O_2_/N_2_ selectivity (as discussed below).

**Table 5:** Calculated useable surface coverages (Δ*θ*) and gravimetric and volumetric O_2_ working capacities for nine frameworks discussed in the text as implemented in VTSA process with *P*_ads_ = 0.21 bar O_2_ at 298 K and *P*_des_ = 0.01 bar at 338 K. Gravimetric working capacities (mmol g^−1^) were obtained by multiplying Δ*θ* by estimated gravimetric O_2_ capacities for each MOF, determined in most cases from the inflection point of the experimental isosteric heat of adsorption as a function of loading (see Section S5 of the ESI for details).^a^ Volumetric working capacities (g L^−1^) were calculated by multiplying Δ*θ* by the estimated volumetric O_2_ capacity for each framework (see Table S12 for details)

MOF	Surface coverage, Δ*θ*	Working capacity (mmol g^−1^)	Working capacity (g L^−1^)
*Co-BTP*	*0.86*	*2.4*	*78.0*
Cu^I^-MFU-4*l*	0.71	1.1	20.3
Co-BDTriP	0.43	0.90	29.2
Mn-PCN-224	0.52	0.29	4.3
Co-BTTri	0.083	0.23	6.8
Fe-BTTri	0.095	0.22	6.2
Fe-PCN-224 HT	0.088	0.048	0.77
Co-PCN-224	0.068	0.048	0.75
Co_2_(OH)_2_(bbta)	0.014	0.035	1.2
Fe-PCN-224 LT	0.039	0.021	0.34

a The inflection point was approximated from the second derivative of the enthalpy *versus* loading, estimated using the finite difference method.

### 3.6 O_2_ working capacities and selectivities

#### 3.6.1 Working capacities

The theoretical O_2_ capacity of an adsorbent, as determined from the density of open metal sites in the MOFs discussed here, is a property inherent to the material. In contrast, the actual working capacity of the framework will also depend on the process conditions. In general, the greater the working capacity of the material, the lower the operating and capital costs of the adsorptive process. Specifically, increasing gravimetric working capacity (units of mmol g^−1^, for example) can increase the product throughput, defined as kg of O_2_ separated per kg adsorbent per hour, whereas increasing volumetric working capacity (units of g L^−1^, for example) can decrease the necessary contactor size—or the size of the system that contains the adsorbent bed—and therefore capital costs.^12^

To estimate the working capacities of the nine investigated frameworks under the aforementioned VTSA process conditions—O_2_ adsorption at 298 K and 0.21 bar/desorption at 10 mbar and 338 K—we first determined an estimated gravimetric O_2_ capacity in each case (in units of mmol g^−1^), based on the inflection point in the plot of enthalpy of adsorption as a function of loading (see the ESI Section S5 for details and Table S12†). The gravimetric working capacity in each case was then calculated as the product of this estimated capacity and the process-specific Δ*θ* value for each MOF. Separately, volumetric working capacities (in units of g L^−1^) were calculated as the analogous product of Δ*θ* and the estimated volumetric capacity in each case. Each estimated volumetric capacity was obtained as the product of the theoretical volumetric capacity (calculated from the single-crystal density) and the multiplicative factor [estimated capacity (mmol g^−1^)]/[theoretical capacity (mmol g^−1^)] (see Table S12† for details; note that the volumetric density of a pellet might differ substantially from the single-crystal value). Table 5 summarizes the resulting gravimetric and volumetric working capacities for each framework in the VTSA process. The materials Cu^I^-MFU-4*l* and Co-BDTriP exhibit the highest working capacities of the eight reported frameworks considered, although these capacities are relatively low (∼1 mmol g^−1^). Interestingly, the gravimetric working capacity of *Co-BTP* is predicted to be 2.4 mmol g^−1^. Under the VSA process conditions described earlier, Cu^I^-MFU-4*l* and Co-BDTriP again exhibit the highest volumetric and gravimetric working capacities of the reported materials (see Fig. S2 and Tables S13, S14†). It is noteworthy that the useable surface coverage and gravimetric and volumetric working capacities of those two frameworks are largest even though their associated enthalpies of O_2_ binding are not the highest of the materials considered. Ultimately, these data emphasize the importance of considering both Δ*H* and Δ*S* when evaluating potential material targets.

We selected Cu^I^-MFU-4*l*, Co-BDTriP, and *Co-BTP* to further investigate the effects of desorption temperature on volumetric working capacities and useable surface coverages in a VTSA process involving adsorption of air at 298 K and 1 bar (0.21 bar O_2_) and desorption at 0.2 bar (see Fig. S3 and S4,† respectively). The working capacities of Cu^I^-MFU-4*l* and Co-BDTriP increase with increasing desorption temperature until they begin to plateau above 370 K, reaching values of ∼21 and 29 g L^−1^, respectively, at 418 K. In the case of *Co-BTP*, the working capacity is projected to increase with temperature up to at least 418 K, the highest temperature considered. As might be expected, the useable surface coverage of each material also generally increases with increasing desorption temperature (Fig. S4†). Interestingly, at the lowest desorption temperatures, Cu^I^-MFU-4*l* and Co-BDTriP exhibit slightly higher working capacities and useable surface coverages than *Co-BTP*, whereas *Co-BTP* outperforms both frameworks at the highest desorption temperatures.

#### 3.6.2 O_2_/N_2_ selectivities

The rigorous determination of adsorbent selectivity for one component of a complex mixture, such as O_2_ from air, requires the collection of multi-component adsorption data. One approach is to conduct breakthrough experiments, which require a large amount of sample and dedicated instrumentation. However, reasonable selectivity estimates can be obtained using single-component adsorption data and Ideal Adsorbed Solution Theory (IAST).^142^ This theory extends Raoult's Law to the adsorbed-gaseous equilibrium and assumes that the adsorbed phase will behave like an ideal solution; IAST has been shown to be accurate across a wide range of adsorbents and gas mixtures.^143,144^ However, it is important to note that a redox-mediated adsorption mechanism may result in a non-negligible thermodynamic change in the adsorbent, in which case the assumptions of IAST may not fully hold. In these cases, more accurate assessments of adsorption selectivity can be gained from Monte Carlo simulations.^145–147^ Experimentally, adsorption selectivities can also be measured using *in situ* Fourier transform IR^148^ and NMR^149^ spectroscopies, mass spectrometry coupled with volumetric assays,^150^ and multi-component equilibrium adsorption measurements.^151^

Table 4 includes the reported O_2_/N_2_ IAST selectivities for several frameworks for a 21 : 79 O_2_/N_2_ mixture at 1 bar. Because these values are reported at different temperatures, it is difficult to draw precise comparisons. Nonetheless, we note some key takeaways. For instance, the selectivity of Co-BDTriP is more than double that of Co-BTTri at 195 K, highlighting the power of ligand modifications to tune adsorption properties. However, the most practical materials will be those that are highly selective for O_2_ at ambient temperature or above.^14,152^ Cr-BTT displays the highest 298 K IAST selectivity of all the materials examined, although it is not entirely stable to repeated cycling.^123^ Calculated 298 K IAST values for Cu^I^-MFU-4*l* and Co-BDTriP, which exhibit the highest calculated capacities at 298 K, are 6.5 and 14, respectively, for a 21 : 79 O_2_/N_2_ mixture at 1 bar (Fig. S5 and S6†). Note that these values are based on isotherm data collected at lower temperatures (203 to 233 K), and it will be important to validate these estimates experimentally. Even still, based on all the metrics evaluated above, these two MOFs clearly stand apart from the rest as the most promising O_2_-selective adsorbents, and it may be worthwhile to pursue synthetic variants that exhibit further optimized O_2_ adsorption properties suitable for practical applications.

### 3.7 O_2_ adsorption without metal coordination

We conclude Section 3 with a brief overview of some additional frameworks that have been shown to selectively bind O_2_*via* mechanisms that do not involve redox-active open metal sites. As only a small fraction of frameworks studied to date feature such sites, it is of interest to explore alternative strategies for achieving selective O_2_ uptake in metal–organic frameworks.

#### 3.7.1 Outer-sphere electron transfer

Recently it was shown that reduced frameworks of the type A_*x*_Fe_2_(bdp)_3_ (A = Na^+^, K^+^; bdp^2−^ = 1,4-benzenedipyrazolate; 0 < *x* ≤ 2)^153^ are capable of selectively binding O_2_ over N_2_ at ambient (25 °C) or even elevated (200 °C) temperatures.^152^ These mixed-valence materials are prepared *via* chemical reduction of the parent Fe_2_(bdp)_3_ with alkali naphthalenides and feature one-dimensional pyrazolate-bridged chains of coordinatively-saturated iron centers. Notably, O_2_ uptake in these materials occurs due to *outer-sphere* electron transfer, in contrast to the MOFs discussed above, and the resulting superoxide moieties are stabilized by alkali cations residing within the pores. A suite of structural and spectroscopic data indicate that superoxide formation is associated with significant rearrangement of the alkali cation positions. This phenomenon is kinetically limiting at ambient temperature, precluding the use of these materials for practical applications. However, the use of larger, templating cations that would promote O_2_ reduction may be a means of enhancing cyclability. Ultimately, these results represent an important proof-of-concept of the utility of outer-sphere electron transfer for promoting strong, selective O_2_ binding and motivate further pursuit of chemically or electrochemically reduced frameworks for selective O_2_ binding.

#### 3.7.2 O_2_ binding at redox-active linkers

Metal–organic frameworks can also be synthesized with redox-active organic linkers. If these linkers are sufficiently reducing and not sterically encumbered, they may also serve as O_2_ binding sites. Few examples of reversible O_2_ binding to organic molecules exist,^154,155^ and to our knowledge only one example exists in a framework. In particular, the flexible, porous coordination solid comprised of Zn-4,4′-bipyridyl chains bridged by TCNQ (7,7,8,8-tetracyano-*p*-quinodimethane) dimers was shown to bind O_2_ at 77 K.^156^ Although the adsorption data were obtained below the boiling point of O_2_ and are therefore not directly relevant to the separation of O_2_ from air, these results are nonetheless intriguing. Indeed, *in situ* IR and Raman spectroscopy data suggest partial charge transfer from the TCNQ dimers to O_2_. It may be possible to access more reduced O_2_ species in MOFs, and at higher temperatures, through the use of more reducing linkers. For example, electrochemically reduced trinitroarene molecules have been shown to reversibly bind O_2_ as μ-O_2_^2−^ species.^154,155^

#### 3.7.3 Additional binding modalities

A small number of materials have been reported to selectively bind O_2_ over N_2_ through other diverse mechanisms. We highlight a few examples here, although in most cases the selectivity for O_2_ is not fully explained. The framework Sc_3_(btc)_2_ exhibits slight selectivity for O_2_ over N_2_ at 298 K, which was attributed to framework flexibility and a purported favorable binding pocket for O_2_.^157^ The microporous metal formate Mn(HCOO)_2_ has also been shown to selectively adsorb O_2_ over Ar and N_2_ below 140 K, although the mechanism is not well-understood.^158^ Finally, a family of multicomponent frameworks featuring triangular Cu^I^_3_(HPyC)_3_ (HPyC^−^ = 4-pyrazolecarboxylate) units have been shown to undergo facile oxidation in the presence of water.^159^ Upon oxidation, the linear copper(i) centers are converted to square planar copper(ii) with coordination spheres completed by a terminal hydroxide and μ^3^-OH. This reaction is reversible with heating under vacuum or treatment with a mildly reducing solvent. This mechanism is clearly distinct from the other modes of O_2_ binding at transition metal sites discussed above, and it is interesting to consider, as a more general strategy, the use of water or other small molecules to facilitate reversible O_2_ adsorption in MOFs.

## 4. Density functional theory calculations of O_2_ binding in MOFs

### 4.1 DFT-based methods for predicting O_2_ binding energies in MOFs

Density functional theory-based computational studies of O_2_ binding in MOFs can play an important role in supporting experimental design and characterization efforts, and in advancing understanding of binding mechanisms. In general, it can be a challenge to predict the nature of small molecule binding at coordinatively-unsaturated, open-shell transition metals in MOFs using contemporary DFT methods, given that unit cells may contain hundreds of atoms, and because standard functionals can fail to adequately treat exchange and correlation effects associated with van der Waals dispersion and states having open-shell spin and localized d orbital character. Previous studies that have predicted energies associated with O_2_ binding to open metal sites in MOFs have used DFT calculations that either treat the full crystalline system with periodic boundary conditions^160–162^ or focus on the local O_2_ binding environment using cluster models.^44,163,164^

MOFs are highly ordered, crystalline materials that can be modeled using periodic boundary conditions, and periodic DFT calculations, typically carried out using an entire unit cell, can enable a realistic description of the framework structure. Important onsite correlation effects—associated with redox-active transition metal centers with open d shells—can be addressed with periodic DFT calculations using hybrid functions or semi-empirical Hubbard *U* corrections (so-called “DFT + *U* calculations”),^161^ which act on the d-states localized on the metal centers. Although empirical in nature and approximate, Hubbard *U* corrections can lead to improved treatments of electron–electron interactions and are less computationally expensive than using hybrid functionals on a MOF with a large unit cell. It should be noted that prior calculations of energies associated with O_2_ (ref. 160 and 161) and CO_2_ (ref. 40 and 165) binding in MOFs using DFT + *U* have shown that the energies can increase or decrease monotonically with increasing *U*, depending on the nature of binding. While it is possible to obtain Hubbard *U* values with a first-principles approach, such values do not always improve agreement with experiment.^158,162^

Cluster calculations, where only a small number of atoms near the binding site are treated explicitly, can reduce computational complexity while allowing for a more accurate treatment of open-shell systems, through more efficient use of hybrid functionals or more rigorous treatment of local interactions, for example through the use of so-called higher-rung density functionals or even beyond-DFT wave-function based quantum chemistry approaches. However, care must be taken when choosing this approach, as cluster calculations do not consider long-range interactions, which may be important in influencing experimental O_2_ binding properties. Relatedly, cluster calculations typically “lock in” the positions of certain atoms, fixing them to their periodic bulk positions, to prevent atomic displacements that would be unfeasible or unrealistic in the extended MOF system. If the initial constrained bond lengths are unfeasibly large (or small) based on the experimentally known spin state for an O_2_-bound metal site, it can be difficult to converge the calculation to the correct spin state. Knowledge of the metal–ligand bond lengths alone leaves some ambiguity for how one ought to fix the atomic positions in the cluster. This ambiguity is less of an issue when performing calculations with periodic boundary conditions, where the atomic positions and lattice parameters can relax with greater freedom. For scenarios where calculations on truncated MOF clusters are desirable due to the aforementioned advantages, a periodic DFT calculation at a lower level of theory can first be performed to establish reasonable bounds for variations in bond lengths.^44^

### 4.2 DFT calculations of O_2_ binding in M–benzenetrisazolates

In this section, we describe the results of DFT calculations performed using clusters and periodic boundary conditions to determine predicted O_2_ binding energies in the M-BTT, M-BTTri, and hypothetical *M-BTP* frameworks (M = Cr^II^, Mn^II^, Fe^II^, Co^II^). The M–benzenetrisazolates were chosen for this purpose as they represent the largest and most thoroughly investigated family of materials studied to date for O_2_ capture and exhibit promising capacities, adsorption enthalpies, and metal site densities.

**Fig. 5:**
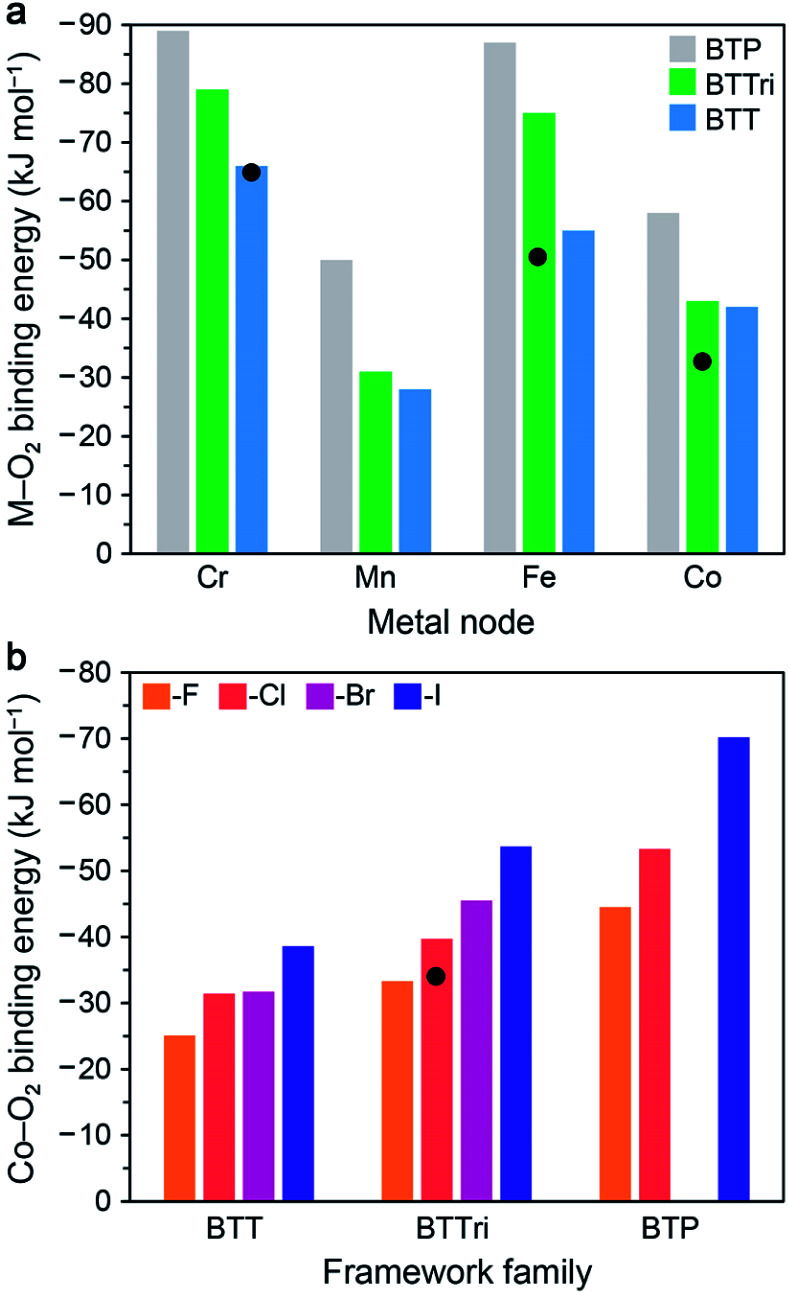
(a) Results of DFT cluster calculations for O_2_ binding energies in a series of model four-metal clusters [M_4_Cl(azolate)_8_]^−^ (M = Cr^II^, Mn^II^, Fe^II^, and Co^II^; azolate = pyrazolate, triazolate, or tetrazolate to represent BTP^3−^, BTTri^3−^, or BTT^3−^ linkers). The TPSSh functional was used for M = Mn^II^, Fe^II^, and Co^II^, and M06 was used for Cr^II^ (see Section S7.2 of the ESI† for details). Experimental enthalpy values are shown as black circles for Cr-BTT, Fe-BTTri, and Co-BTTri. As can be seen, TPSSh tends to overestimate experimental binding energies. Binding energies trend with the basicity of the azolate across all metals. (b) DFT calculations with periodic boundary conditions (PBE-D3 + *U*, *U* = 3.3 eV) for O_2_ binding energies for the series of [(Co_4_X)_3_(benzenetrisazolate)_8_] (X = F^−^, Cl^−^, Br^−^, and I^−^; benezentrisazolate = BTT^3−^, BTTri^3−^, and BTP^3−^). The experimental O_2_ binding enthalpy for Co-BTTri is shown as a black circle. Notably, binding energies trend with the electropositivity of the halide.

For DFT cluster calculations, we used the four-metal cluster [M_4_Cl(azolate)_8_]^−^ to represent the local binding site and the TPSSh hybrid functional (see Fig. S7 and ESI Section S7.2† for details). As shown in Fig. 5a, our DFT calculations predict an increase in the O_2_ binding strength with increasing basicity of the azolate linker for all four metals considered, consistent with available experimental data. The calculated O_2_ binding energies for the model clusters for Fe-BTTri, and Co-BTTri are larger than the experimentally determined enthalpies for the frameworks, but we note that the degree of over- or under-estimation is heavily influenced by the choice of exchange-correlation functional (see Table S18†). Overall, our calculations suggest that Mn-BTP, Fe-BTT,^125^ and *Co-BTP* may be promising materials for O_2_ separations, given that their predicted binding energies are near the proposed optimal binding enthalpy of −45 kJ mol^−1^ (refer to Section 3.1). We note that while the synthesis of Co-BTT has been reported,^127^ its O_2_ adsorption properties have not been investigated. Based on these calculations and the experimental enthalpies of O_2_ adsorption in the congeners with more basic ligands (Co-BTTri and Co-BTTriP), Co-BTT is not likely of interest for further study related to air separations.

Our calculations of O_2_ binding to open metal sites in the M–benzenetrisazolate frameworks were carried out using periodic boundary conditions with an eye toward identifying other isostructural materials that may display O_2_ binding enthalpies near the proposed optimal value of −45 kJ mol^−1^. We started with the experimentally determined structure for Co-BTTri and replaced the linker or halide to obtain the various structures considered. Calculations were performed on unit cells of the type [(Co_4_X)_3_(benzenetrisazolate)_8_] (X = F^−^, Cl^−^, Br^−^, and I^−^; benezentrisazolate = BTT^3−^, BTTri^3−^, and BTP^3−^) without charge balancing cations. As such, to represent the anionic framework accurately with an overall neutral unit cell, we added three extra electrons and a positive neutralizing background charge (see ESI Section S7.1† for details). All of the atoms and the lattice vectors were then relaxed. We performed all DFT calculations with PBE + *U*, and we also employed a pairwise correction term, Grimme D3, to capture van der Waals dispersion corrections. These periodic DFT + D3 + *U* calculations ultimately enabled us to perform full geometry optimizations without constraining the positions of the atoms, as is necessary in the cluster calculations.

As shown in Fig. 5b, these calculations predict that the O_2_ binding energy increases upon moving from X = F^−^ to I^−^. This result may be explained by considering that the electron density around the cobalt sites will increase with the electropositivity of the μ^4^-halide, and therefore charge transfer to O_2_ would be increasingly favored. The results further suggest that Co_3_[(Co_4_Br)_3_(BTTri)_8_]_2,_ Co_3_[(Co_4_I)_3_(BTTri)_8_]_2_, Co_3_[(Co_4_F)_3_(BTP)_8_]_2_, and Co_3_[(Co_4_Cl)_3_(BTP)_8_]_2_ may be promising target materials. While the absolute binding energies will be dependent upon the choice of exchange–correlation functional, including the choice of the method used to treat dispersion interactions and the Hubbard *U* value chosen, it is expected that trends found with a given functional should hold for other choices of functional. Thus, since Co_3_[(Co_4_Cl)_3_(BTTri)_8_]_2_ has an experimental binding energy below the target value of −45 kJ mol^−1^, based on our calculated trends, we would expect that Co_3_[(Co_4_X)_3_(BTTri)_8_]_2_ with X = Br or I would have binding enthalpies close to −45 kJ mol^−1^. Since no members of the Co_3_[(Co_4_X)_3_(BTP)_8_]_2_ series have yet been synthesized, whether any of its variants would have a binding enthalpy near −45 kJ mol^−1^ is unclear, although we would expect from our trends that Co_3_[(Co_4_Cl)_3_(BTP)_8_]_2_ would bind O_2_ more strongly than Co_3_[(Co_4_Cl)_3_(BTTri)_8_]_2_ and that substituting chloride with larger halides would increase the binding enthalpy.

### 4.3 Benchmarking entropic contributions to O_2_ binding in MOFs

While the calculation of O_2_ binding enthalpies is important for evaluating candidate adsorbents, the free energy of adsorption enables a more holistic evaluation of material performance, as discussed in detail above. Obtaining vibrational properties from calculations allows one to additionally calculate Δ*S* and estimate the Δ*G* of adsorption. With the calculated free energy in hand, one can also estimate the adsorption isotherm from an *ab initio* calculation. In addition, vibrational calculations provide the O–O stretching frequency, an important observable for determining the degree of charge transfer to dioxygen.

Given the predominant focus in the experimental literature on the enthalpy of O_2_ binding in candidate MOFs, computational efforts have also often focused on this thermodynamic variable. To support future computational and experimental efforts in this area, we investigated how accurately cluster calculations can estimate the Δ*S* of O_2_ binding for the systems of interest here. We chose the M-PCN-224 (M = Mn^II^, Fe^II^, Co^II^) family as a model system because (i) their experimental O_2_ binding entropies span a wide range of values (see Fig. 4a) and (ii) the cubic pore shape, face-centered location of the porphyrins (see Fig. 3f), and pore size (approximately 19 Å) are such that one may less ambiguously select the relevant cluster—in this case the metal–porphyrin molecule—when compared to the M-BTTri series.

We first examined various functionals for their accuracy in predicting binding energy. In the case of Mn-porphyrin, many commonly used functionals, even ones benchmarked to transition metal datasets (*e.g.*, MN15, ωB97X-D, M06, PBE0),^166–169^ failed to predict the peroxide species bound to Mn(ii) (see ESI Section S7.3 for details and Table S20†). Both TPSSh and B97M-rV yielded good estimates of binding energies, but B97M-rV requires far more expensive frequency calculations. Ultimately, TPSSh correctly identified the peroxide and superoxide species that form upon O_2_ binding in Mn- and Fe-porphyrin, respectively. The calculated entropy of O_2_ binding in Mn-porphyrin is very close to the value calculated for Mn-PCN-224 using the Clausius–Clapeyron relationship (−179 *vs.* −174 ± 20 J mol^−1^ K^−1^, respectively, see Tables S20 and S11†). Similarly, the calculated entropy of O-_2_ binding in Fe-porphyrin is −143 J mol^−1^ K^−1^, consistent with the value of −121 ± 30 J mol^−1^ K^−1^ determined for Fe-PCN-224 at low temperature (Tables S21 and S11†). In contrast, TPSSh did not identify the superoxide species bound in Co-porphyrin, and the calculated O_2_ binding entropy is much larger than that determined for Co-PCN-224 (−139 *vs.* −59.6 ± 0.7 J mol^−1^ K^−1^, respectively).

## 5. Conclusions and outlook

As a result of their high surface areas and atom-level tunability, metal–organic frameworks have emerged as promising candidates for O_2_-selective adsorptive air separations. In particular, a number of MOFs featuring coordinatively-unsaturated redox-active metal sites have been discovered to date that are capable of selectively binding O_2_ over N_2_. Additional strategies for selective O_2_ adsorption have also begun to emerge, such as outer-sphere electron transfer from redox-active frameworks and non-redox-mediated chemisorption of O_2_. Overall, the continued investigation of new MOFs and O_2_ adsorption mechanisms is a key fundamental driver of this important, nascent area of research.

With an eye toward practical applications, frameworks with open metal sites are the most promising materials studied to date. Design strategies have focused on tuning the linker and local coordination environment to generate open metal sites that are sufficiently reducing for selective O_2_ binding and can be regenerated using relatively mild swings in temperature or pressure. However, significant improvements are still needed to render MOFs competitive with the incumbent adsorptive air separation technology based on nitrogen-selective zeolites.

Drawing on inspiration from molecular and biological systems that strongly and reversibly bind O_2_, we have identified design considerations for further enhancing selective, reversible O_2_ uptake in MOFs (Section 2), surveyed key frameworks studied to date for selective O_2_ uptake (Sections 3.2–3.4), and evaluated relevant performance metrics (Sections 3.5 and 3.6). While the enthalpy of O_2_ binding has traditionally been used to judge material performance, it is the free energy of adsorption, Δ*G*, that is most important in evaluating suitability for a practical separation process. Thus, going forward, it will be critical for researchers to adopt a more holistic approach, considering both the enthalpy and entropy of O_2_ binding in the evaluation of any new MOF for air separations. Other important parameters to consider are gravimetric and volumetric O_2_ capacities, based on exposed metal site density, which can readily be estimated from experimental data.

For new frameworks, it will be critical to characterize these properties under working conditions relevant for practical separations, and ambient temperature data should be reported when possible. More rigorous characterization will in turn enable a greater fundamental understanding of O_2_ binding at open metal sites. Never has it been so facile to determine thermodynamic parameters for O_2_ binding across isostructural series, as exemplified in the case of the M-PCN-224 (M = Mn^II^, Fe^II^, Co^II^) frameworks. Importantly, this understanding will also carry over to other fields working on O_2_ activation and reactivity.^170^

The free energy of O_2_ binding can ultimately be used to determine optimal working conditions for a given adsorbent, or alternatively, to identify an optimal adsorbent for a given set of working conditions. Using available experimental data, we put the latter concept into practice and evaluated the performance of nine MOFs in representative vacuum swing and vacuum/temperature swing adsorption processes. Two known MOFs, Cu^I^-MFU-4*l* and Co-BDTriP, and the hypothetical material *Co-BTP*, stood out from this analysis as top performers. Beyond this practical insight, these results also suggest that design iterations based on the former two frameworks (and pursuit of *Co-BTP*) are promising research directions.

Finally, it is important to note that the guidelines established here encompass only material-level factors that can be tuned to accelerate the development of O_2_-selective MOFs. Beyond initial design, characterization, and identification of promising candidates, numerous system level factors will also be key to consider in prioritizing materials, such as pellet density, thermal conductivity, cost of synthesis, and impurity resilience. In all, there is a wealth of chemistry yet to be explored in the development of MOFs for selective O_2_ capture.

## Author contributions

D. E. J, T. D. H., and J. R. L. conceived the manuscript. D. E. J., A. J., B. E. R. S., A. S., E. T., R. R., M. N. D., W. D., J. B. N., T. D. H., and J. R. L. prepared and contributed to portions of the manuscript. K. R. M. provided a critical review and contributed to revisions.

## Data availability

The datasets supporting this article have been uploaded as part of the ESI.†

## Conflicts of interest

There are no conflicts to declare.

## Supplementary Material

SC-013-D2SC03577D-s001

SC-013-D2SC03577D-s002
