# Supplementary material for: Metal–organic frameworks as O2-selective adsorbents for air separations
Source: Chem Sci. 2022 Aug 11;13(35):10216–37. doi: 10.1039/d2sc03577d (PMC9473493; doi:10.1039/d2sc03577d)
Supplement: SC-013-D2SC03577D-s002 [file SC-013-D2SC03577D-s002.pdf]

Electronic Supplementary Material (ESI) for Chemical Science.
This journal is © The Royal Society of Chemistry 2022

Supplementary Information for:

Metal–organic frameworks as O_2-selective adsorbents for air separations

David E. Jaramillo,[a] Adam Jaffe, [a] Benjamin E. R. Snyder, [a] Alex Smith,[b] Eric Taw,[c,d] Rachel Rohde,[a] Matthew N. Dods,[a] William De Snoo,[b] Katie R. Meihaus,[a] T. David Harris,[a] Jeffrey B. Neaton, [b,e,f] Jeffrey R. Long*[a,c,d]

a. Department of Chemistry, University of California Berkeley, Berkeley, California 94720, USA; Email: jrlong@berkeley.edu

b. Department of Physics, University of California Berkeley, Berkeley, California 94720, USA

c. Department of Chemical and Biomolecular Engineering, University of California Berkeley, Berkeley, California 94720, USA.

d Materials Science Division, Lawrence Berkeley National Laboratory, Berkeley, California 94720, USA

e. Molecular Foundry, Lawrence Berkeley National Laboratory, Berkeley California, 94720, USA

f. Kavli Nanosciences Institute at Berkeley, Berkeley, California, 94720, USA

Chem. Sci.

Table of Contents

Section S1. Minimum work calculation 2
Section S2. Comparison of energy demand between N_2- and O_2-selective adsorbents 3
Section S3. Estimate of entropic contributions to O_2 binding 4
Section S4. Adsorption isotherm fitting 5
Section S5. Differential enthalpies and entropies of adsorption 6
Section S6. Calculation of ΔG for VSA and VTSA model processes 7
Section S7. DFT calculations of O_2 binding in MOFs 8
Figures 12
Tables 18
References 29

Section S1. Minimum Work Calculations

By combining the first and second laws of thermodynamics, one can establish a useful framework for assessing the theoretical energy requirement for a separation process. The minimum separation work for a multicomponent stream of ideal gases (e.g., O_2 and N_2 at near-ambient temperature and pressure) can be written as follows:

$$W_{\text{min}} = -RT \sum_i x_i \ln(x_i)$$

where x_i is the mole fraction of component i prior to separation, and T and R are the stream temperature and ideal gas constant, respectively.[1] The above equation can be understood as the minimum energy needed to perfectly separate all of the components i from the mixture, at constant temperature and pressure. For simplicity, we describe air as an ideal mixture of only three components, N_2, O_2, and Ar, implying:

$$W_{min} = -RT[x_{O2} \ln(x_{O2}) + x_{N2} \ln(x_{N2}) + x_{Ar} \ln(x_{Ar})]$$

Note that this expression for W_{min} computes the minimum work per *total* moles of gas. If O_2 is taken as the desired component, the minimum work per mole of O_2 can be computed simply from:

$$W_{min,O2} = -\frac{RT}{x_{O2}}[x_{O2} \ln(x_{O2}) + x_{N2} \ln(x_{N2}) + x_{Ar} \ln(x_{Ar})]$$

Clearly, $W_{min,O2}$ varies linearly with temperature. Although cryogenic distillation implies sub-ambient temperatures, these temperatures must be generated *via* allocation of usable work, and thus the calculation of $W_{min,O2}$ must be interpreted in light of the lifecycle exergy balance. In order to enable a parallel comparison to adsorptive N_2/O_2 separations, which often operate at near-ambient temperatures, we select T = 25 °C for all calculations. Taking $x_{N2} = 0.78$, $x_{O2} = 0.21$, and $x_{Ar} = 0.01$, we obtain $\boldsymbol{W_{min,O2}}$ **= 6.7 kJ/mol O_2**. This value can be compared across different separation technologies (e.g., cryogenic distillation and pressure-swing adsorption) to assess the energy efficiencies of each approach. For example, cryogenic distillation commonly requires 200 kWh/tonne O_2, which is ~3.5 times larger than $W_{min,O2}$.

In addition to comparing actual work expenditures to thermodynamic minima, it is crucial to recognize the distinction between separation work and primary energy. For example, the electricity used to power compressors and pumps in cryogenic distillation is typically supplied from the combustion of fossil fuels. The efficiency of the conversion between primary energy (combustion heat) and usable work (electrical energy) must be carefully considered when comparing different N_2/O_2 separations.

Section S2. Comparison of energy demand between N_2- and O_2-selective adsorbents

As a simple estimate, assume that the energy consumption of a given adsorption process is directly correlated with the energy required to regenerate the adsorbent.[1] We can use the negative of the enthalpy of adsorption as a proxy for the required desorption energy. As such, enthalpies of adsorption enable a crude comparison of energy demands across various adsorption processes. In numerous examples, it has been shown that ΔH of adsorption represents about half of the total energy required for regeneration.[2] Consider that Li-LSX exhibits an N_2 binding enthalpy of −22.5 kJ/mol.[3] For every 1 mole of air that is separated, 0.78 mol N_2 × 22.5 kJ/mol = 17.55 kJ are required to regenerate the material. Alternatively, assume an O_2-selective material exhibits an O_2 binding enthalpy of −45 kJ/mol. For every 1 mole of air that is separated, 0.21 mol O_2 × 45 kJ/mol = 9.45 kJ are required to regenerate the material. Thus, in this example, the O_2-selective material is about twice as energy efficient in separating O_2 from N_2.

Section S3. Estimate of entropic contributions to O_2 binding

The entropy of O_2 binding in an adsorbent, ΔS, is dominated by the loss of the two rotational and three translational degrees of freedom of gaseous O_2, which become low-frequency vibrations upon binding (see Figure S1).[4] Smaller contributions arise from changes in the spin state of the metal and bound O_2 species. The change in O_2 entropy upon binding can be estimated from a sum of terms shown in eq. 1–5.

$$S_{rot} = Rlog\left[\frac{e^{3/2}T}{\sigma\Theta_{rot}}\right] \quad (1)$$

Eq. 1 is the molar rotational entropy of a rigid diatomic rotor, where R is the ideal gas constant, T is the temperature, σ is the symmetry number (2 for a homonuclear diatomic), and Θ_{rot} is the rotational temperature (2.08 K for O_2).

$$S_{trans} = Rlog\left[\frac{k_BT}{P\Lambda^3}\right] + 5R/2 \quad (2)$$

Eq. 2 is the Sacktur-Tetrode equation for molar translational entropy of an ideal gas, where R is the ideal gas constant, T is the temperature, k_B is the Boltzmann constant, P is the pressure, and Λ is the thermal wavelength (1.79×10^{-11} m for O_2 at 298 K).

$$S_{elec} = Rlog[S(S+1)] \quad (3)$$

Eq. 3 is the molar electronic entropy due to spin degeneracy, where S is the total spin of the system, operating under the assumption that the spin sublevels are approximately degenerate (i.e., split by an amount that is small relative to k_BT).

$$S_{vib} = R\frac{\theta_v}{T}\frac{1}{e^{\Theta_{vib}-1}} - Rlog[1 - e^{\Theta_{vib}}] \quad (4)$$

Eq. 4 is the molar vibrational entropy of a harmonic oscillator, where $\Theta_{vib} = \hbar\omega/k_B$ is the vibrational temperature, with $\hbar\omega$ the vibrational energy.

$$S_{config} = Rlog[\sigma_{O_2}\sigma_M] \quad (5)$$

Lastly, eq. 5 is the molar configurational entropy due to indistinguishable binding modes, where σ_{O_2} is the symmetry number of O_2 (2 for a homonuclear diatomic), and σ_M is the symmetry number of the metal center. As an example, O_2 binding to a D_{4h} symmetric heme corresponds to a symmetry number of $\sigma_{O_2}\sigma_M = 16$.

The overall expression for the molar entropy change upon binding is then given as eq. 6, where the sum is taken over the five vibrational modes of the MO_2 unit highlighted in Figure S1:

$$\Delta S = \left(S_{elec}(MO_2) + \sum_{i=1}^{5} S_{vib,i}(MO_2) + S_{config}\right) - \left(S_{rot}(O_2) + S_{trans}(O_2) + S_{elec}(O_2) + S_{elec}(M)\right) \quad (6)$$

Section S4. Adsorption isotherm fitting

Low-pressure isotherms were fit with a dual-site Langmuir–Freundlich equation (eq. 7), where n is the total amount adsorbed in mmol/g, P is the pressure in bar, $n_{sat,i}$ is the saturation capacity in mmol/g, b_i is the Langmuir parameter in bar^{-1} defined in eq. 2, and v_i is the Freundlich parameter for each site.[5]

$$n = \frac{n_{sat,1} b_1 P^{v_1}}{1+b_1 P^{v_1}} + \frac{n_{sat,2} b_2 P^{v_2}}{1+b_2 P^{v_2}} \tag{7}$$

$$b_i = e^{S_i/R} e^{\frac{-E_i \cdot 1000}{RT}} \tag{8}$$

For eq. 8, S_i is the site-specific entropy of adsorption in units of J/(mol·K), E_i is the enthalpy of adsorption in units of kJ/mol, R is the ideal gas constant in units of J/(mol·K), and T is the temperature. In the case of Cu^{I}-MFU-4*l* and Fe-PCN-224, published isotherm data were newly fit to obtain the fit parameters. For the remaining frameworks ($Co_2(OH)_2$(bbta), Fe-BTTri, Co-BTTri, Co-BDTriP, Mn-PCN-224, and Co-PCN-224), the published fit parameters were used directly to determine binding enthalpies and entropies (see Tables S1 to S9). For Li-LSX, the published parameters from Ref. 2 were used to determine a binding enthalpy (Table S10).

Section S5. Differential enthalpies and entropies of adsorption

Using the multi-site Langmuir-Freundlich fits, the isosteric enthalpies of adsorption, ΔH, and entropies of adsorption, ΔS, were calculated using the Clausius–Clapeyron relationship (eq. 9), where R is the ideal gas constant, P is the pressure, and T is the temperature.[6]

$$lnP = -\frac{\Delta H}{R}\left(\frac{1}{T}\right) + \frac{\Delta S}{T} \quad (9)$$

The ΔH and ΔS as a function of loading (mmol/g) curves were generated for the frameworks given in Figure 4 and Table 5 in the main text. The ΔH values reported in Table S11 and Figure 4 were obtained by averaging the first several values of ΔH in the ΔH versus loading curves where the curve was flat or relatively flat ("low-loading"). We note that ΔH values reported in the literature may also be found to be the highest value of ΔH from the enthalpy versus loading curve. This approach may suitable in cases where there is little variation in the magnitude of ΔH at low loadings (e.g., as is the case for Fe-BTTri). In cases where there is more variation in ΔH, it is not always straightforward what loading values to select to obtain an average. We recommend at the very least, when reporting ΔH values that the loading range used be explicitly stated. *Ultimately, the goal is to estimate ΔH and ΔS values that are associated with only the primary binding site.* Note that you cannot average up to the inflection point to obtain ΔH, because doing so will include secondary site adsorption. Consequently, the value of ΔH will be an average of metal site binding and secondary site binding.

The range of loadings associated with the chosen ΔH range was then used to determine an average value of ΔS for each MOF, and ΔG_{298} was calculated from these values. All thermodynamic parameters are reported in Table S11.

The reported "estimated O_2 capacities" as given in Table 5 of the main text and Tables S12–S14 were in most cases obtained by estimating the inflection point in the ΔH versus loading curves based on the second derivative of the enthalpy versus loading as estimated using the finite difference method. *The goal with this value is to estimate the number of metal sites that are accessible to O_2 binding.* Utilizing the inflection point in the ΔH versus loading curve is a well-established approach to determine the number of accessible open sites for a given framework. An alternative approach is to take the capacity data from a single isotherm. However, with the latter approach, one cannot always discern if adsorption is due to metal site binding or if there is substantial secondary site adsorption. It is worth noting the limitations of this approach and the complexity involved in these assumptions. Not all ΔH versus loading curves will have an S-like shape, making it difficult to identify the inflection point. This can occur for frameworks with a low-density of binding sites for which the results can be sensitive to experimental error, for example. This is the case for Co-PCN-224 (see the supporting Excel file, "O2IsothermFits"). For this framework we simply assumed the estimated O_2 capacity to be the metal site density. For Fe-PCN-224, the inflection points were different for the HT and LT data sets. For simplicity, we used the inflection point from the LT data set.

Section S6. Calculation of ΔG for VSA and VTSA model processes

The working surface coverage, $\Delta\theta$, is defined in eq. 10 and 11.[5,7] As shown in eq. 10, $\Delta\theta$ is the difference between the surface coverage under adsorption (θ_{ads}) and desorption (θ_{des}) conditions. The maximal value of $\Delta\theta$ under a given set of working conditions is 1, corresponding to complete coverage of the primary O_2 binding sites upon adsorption ($\theta_{ads} = 1$) and zero coverage upon desorption ($\theta_{ads} = 0$). In eq. 11, P is the pressure in bar, R is the ideal gas constant in units of J/(mol·K), T is the temperature in kelvin, and ΔG is in J/mol. For a given temperature and pressure, there is a value of ΔG that will correspond to maximum $\Delta\theta$.

$$\Delta\theta = \theta_{ads} - \theta_{des} \quad (10)$$

$$\Delta\theta = \frac{e^{\frac{-\Delta G_{ads}}{RT}} P_{ads}}{1 + e^{\frac{-\Delta G_{ads}}{RT}} P_{ads}} - \frac{e^{\frac{-\Delta G_{des}}{RT}} P_{des}}{1 + e^{\frac{-\Delta G_{des}}{RT}} P_{des}} \quad (11)$$

Section S7. DFT calculations of O_2 binding in MOFs

As discussed in the main text, when performing DFT calculations to study O_2 binding in metal–organic frameworks, one can choose to treat the full crystalline system with periodic boundary conditions or to focus only on the local O_2 binding environment in a cluster calculation. Periodic calculations enable a more realistic description of the MOF geometry, because all of the atoms in the calculation have the correct coordination environment, but they are typically computationally more expensive and, in many cases, make it infeasible to do hybrid functional calculations, which can be important for open-shell systems. Cluster calculations require choosing how to truncate the crystalline system to treat it as an isolated molecule, but have the advantage of being able to use hybrid calculations and beyond-DFT methods from quantum chemistry. Below, we show that both PBE+*U* calculations with periodic boundary conditions and cluster calculations with hybrid meta-GGA functionals well capture trends in O_2 binding energetics for MOFs with available experimental binding energies to use as a reference. However, one of the materials, Fe-BTTri, undergoes a spin state transition upon O_2 binding, and PBE+*U* was not able to yield the correct spin state before and after O_2 binding with the same Hubbard *U* value. The cluster calculations succeeded in giving the spin transition in Fe-BTTri, highlighting the advantage that hybrid functionals provide in capturing the properties of open-shell systems. Additionally, we use the cluster calculations to compare different hybrid and hybrid-meta-GGA functionals.

Section S7.1 M-benzenetriazolates ($M_3[(M_4X)_3(\text{benzenetrisazolate})_8]_2$) periodic calculations. The M-BTTri and Cr-BTT structures contain disordered pore-dwelling cations in order to charge balance the anionic frameworks. Past computational studies using periodic systems have charge balanced the anionic framework by protonating one nitrogen on the azolate per metal node, rather than including such pore-dwelling cations.[8,9] This approach makes geometry optimization converge faster relative to incorporating pore-dwelling cations. On the other hand, the electronic structure of the coordinatively unsaturated metal sites may differ between structures with neutral and anionic linkers. In order to maintain the formal anionic charge on the linkers while excluding pore dwelling cations, we added three electrons to the unit cell $[(M_4X)_3(\text{benzenetrisazolate})_8]$ (M = Cr^{II}, Mn^{II}, Co^{II}; X = F^-, Cl^-, Br^-, I^-; benzenetrisazolate = BTT^{3-}, $BTTri^{3-}$, BTP^{3-}) and introduced a positive neutralizing background charge. We performed DFT geometry optimizations to obtain O_2 binding energies and transition metal spin states for $[(Co_4X)_3(\text{benzenetrisazolate})_8]$ (X = F^-, Cl^-, Br^-, I^-; benzenetrisazolate = BTT^{3-}, $BTTri^{3-}$, BTP^{3-}) and $[(M_4Cl)_3(\text{benzenetrisazolate})_8]$ (M = Cr^{II}, Mn^{II}, Co^{II}) using periodic boundary conditions and a plane-wave basis set. For these calculations, we used the Vienna ab initio Simulation Package (VASP) and the generalized gradient approximation of Perdew, Ernzerhof, and Burke (PBE), with projector augmented-wave (PAW)[10] pseudopotentials.[11–15] Our pseudopotentials included all 3d and 4s electrons for Cr, Mn, and Co, one electron for H, four electrons for C, five electrons for N, six electrons for O, and seven electrons for Cl. Effects of long-range dispersion interactions using the DFT+U approach and D3 corrections[16] with Becke-Johnson damping.[17] We used a Hubbard *U* value with the Dudarev approach[18–20] to approximately treat correlation effects associated with localized d states on the transition metal sites; our *U* values were chosen based on previously-reported transition metal oxide calculations.[21] For $[(M_4X)_3(\text{benzenetrisazolate})_8]$, there are three metal nodes per cubic unit cell, and we considered the case where O_2 binds to all four sites at one metal node. Thus, we can express the binding energy per O_2, ΔE_{O_2}, using eq. 12, where E_{MOF-O_2} is the energy of the framework with bound O_2, E_{MOF} is the energy of the framework, and E_{O_2} is the energy of free O_2.

$$\Delta E_{O_2} = \frac{1}{4}(E_{MOF\text{-}O_2} - E_{MOF} - 4E_{O_2}) \quad (12)$$

Our structural relaxations used a Γ point sampling of the Brillouin zone and a 600-eV plane-wave energy cutoff energy. We used spin-polarized calculations with the initial transition metal spin chosen to match the expected spin state. The unit cell shape, unit cell volume, and the ions were relaxed until the Hellman-Feynman forces were less than 0.01 eV/A. To account for Pulay stress due to a changing cell shape, we looped converged calculations from their last set of lattice vectors and atomic positions until the forces were converged after just a single ionic step. The unit cell contains 231 atoms, and optimized lattice parameters are given in Table 15.

We initialized our unit cell geometry with the reported experimental structure of Co-BTTri[22] and replaced the metal, ligand, or halogen accordingly (see Figure 3e in the main text for the structure of the isostructural Fe-BTTri). An experimental structure is also reported for Cr-BTT,[23] and we computed the energy of O_2 binding in Cr-BTT based on an initialization from those experimental atomic positions and also from the coordinates of Co-BTTri, with Co replaced with Cr and BTTri replaced with BTT. We did this because in the experimental structures of Co-BTTri and Cr-BTT with bound O_2, the O_2 molecules are rotated relative to each other. To study the impact on the binding energy of rotations about the M–O axis, multiple O_2 binding geometries were considered. We performed calculations on Cr-BTT with the O_2 oriented end-on at 0°, end-on at 45°, and end-on at 90°, where the angles are with respect to the plane formed by the metal cluster. Both end-on binding modes at 45° and 90° yielded binding energies of 57 kJ/mol, while the 0° end-on mode was 5 kJ/mol less favorable. These results show that rotation about the M–O bond can affect the binding energy by as much as about 10%.

The results obtained for the O_2 binding energies, metal spin densities, and metal–O_2 bond lengths for [$(M_4Cl)_3$(benzenetrisazolate)$_8$] (M = Cr^{II}, Mn^{II}, Co^{II}) are given in Table S16. The binding energy trends (BTT^{3-} < $BTTri^{3-}$ < BTP^{3-}) are consistent with the results from the cluster calculations (below). For Fe-BTTri, a spin-state transition is expected upon O_2 binding based on results from Mössbauer spectroscopy,[24] in particular, the initially high-spin iron(II) sites transition to low-spin iron(III) upon O_2 binding. However, we found that U values that resulted in a low-spin configuration for Fe in the O_2 bound system did not yield high-spin Fe in the bare framework, and that U values that resulted in high-spin Fe in the bare framework did not yield low-spin Fe in the O_2 bound system. Because the Hubbard U value penalizes double-occupancy of electron orbitals, a higher U value will generally tend to favor high-spin states while a lower U value will tend to favor low-spin states. In order to do a binding energy calculation, one needs to use the same U value for the reactants and products, so our inability to find a U value that was able to yield a spin-state transition prevented us from getting a O_2 binding energy for Fe-BTTri with the experimentally-reported spin-state transition, a limitation of PBE+U. Calculations were also carried out on [$(Co_4X)_3$(benzenetrisazolate)$_8$] (X = F, Cl, Br, I) to examine how changing the bridging halide affects O_2 binding. These calculations followed the methods described above. Starting from the DFT relaxed structure for Co-BTTri, Cl was replaced with F, Br, or I, and the appropriate ligand, and then the resulting structure was relaxed. Our results are summarized in Figure 5b of the main text and Table S17.

Section S7.2 M-azolates ($[M_4X(azolate)_8]^-$) cluster calculations. All cluster calculations were performed with Q-Chem 5.3 using a def2-TZVP basis set and with symmetry turned off. The structure of each cluster was developed from the experimentally determined structure when available, with hydrogens used to truncate the organic linker (see Figure S7). Furthermore, the carbons of the azolates were frozen during geometry optimization. When experimental structures were not available, clusters expected to be low-spin were initialized using Co-BTTri with metals and/or linkers substituted accordingly. Clusters expected to be high-spin were initialized with Fe-BTTri, as one may reasonably expect high-spin 3d metals to have similar geometries to each other. Previous benchmark DFT studies on metal–ligand bond energies do not agree on a single functional that best matches coupled-cluster results or experimental data, indicating that the functional that most accurately reproduces metal–ligand bond energies may vary depending on the system being evaluated. Nevertheless, functionals such as M06, MN15, MN15-L, ωB97M-V, TPSSh, and B97M-rV are recommended most often among these studies.[25–32] We included additional functionals PBE0 and B3LYP given their common use. For functionals without non-local corrections built in (like ωB97M-V or ωB97X-D[33]), we included Grimme D3(BJ). For M06, we used -D3(0).

To decide on a functional to use for all other O_2 binding energy cluster calculations, we tested the functionals listed above against the experimentally determined heat of O_2 adsorption in Co-BTTri (see Table S18). Pure functionals (no exact exchange) like BP86, TPSS, MN15-L, and M06-L dramatically overestimate the binding energy, while functionals with >20% exact exchange tended to underestimate the binding energy. TPSSh, M06, and B3LYP yielded a binding energy within 10 kJ/mol of the experimental heat of adsorption (−34 kJ/mol). We expect vibrational contributions to the energy, should one calculate those, to decrease the magnitude of the binding energy. While M06 gives a heat of adsorption that is numerically closer to the experimental heat of O_2 adsorption in Co-BTTri than TPSSh (−29 versus −43 kJ/mol, respectively), its binding energy is less than the experimentally determined value, and including the above-mentioned vibrational contributions are expected to reduce this value further. For this reason, we moved forward with TPSSh as our functional of choice for the remainder of the calculations (except in the case of Cr, see below).

We used TPSSh to calculate the O_2 binding energy at open metal sites in $[M_4X(azolate)_8]^{2-}$ (M = Co, Fe, Mn) clusters, constructed as above, using Eq. 12 (see Table 19). Consistent with experimental and computational evidence for Co-BTTri,[22] we assumed all metals were antiferromagnetically coupled across the μ_4-halide, and that this configuration is unchanged upon O_2 adsorption. Cobalt was assumed to be low-spin for all clusters, while Mn and Cr were assumed to be high-spin for all clusters. Iron was assumed to be high-spin in all clusters with a transition to a low-spin state upon O_2 adsorption, consistent with what was found for Fe-BTTri.[24] However, we note that the ground state spin configuration for Fe-BTTri was highly dependent on the initial geometry. Using the Co-BTTri model cluster and replacing cobalt with iron while keeping other atoms in the same place before relaxing resulted in either a low- or intermediate-spin structure, as the Co-BTTri M–N bond length is shorter than that found in Fe-BTTri. These bond length differences in the initial structures are important because the azolate carbons are fixed during the relaxation process and cannot move as much as would be required to access the correct geometry for high-spin Fe. Due to numerical issues encountered using TPSSh and our Cr clusters within this study, binding energies for the Cr-based clusters were computed with M06, which were within the range of 60–80 kJ/mol.[23] We expect these values to be qualitatively consistent with those obtained

with TPSSh. All trends found by these cluster calculations are consistent with the results of periodic DFT calculations, that is the binding energy increases as follows: BTT^{3-} < $BTTri^{3-}$ < BTP^{3-}, and Mn < Co < Fe < Cr.

Section S7.3 M-PCN-224 cluster calculations. The M-PCN-224 series is among only a few examples of MOFs featuring four-coordinate, planar metal centers found in the literature.[34–36] Because DFT calculations on molecular porphyrin compounds are common,[37–39] this series of MOFs is an excellent test system for benchmarking functionals. We used molecular porphyrins as a model system to benchmark a select number of functionals to study entropic contributions to O_2 binding. All clusters were obtained from experimentally determined crystal structures of M-PCN-224 by truncating between the porphyrin ring and the rest of the organic linker (see Figure S7). The Mn-porphyrin was assumed to remain high-spin for both the bare structure and the O_2-bound structure. The Co-porphyrin was assumed to remain low-spin. The Fe-porphyrin was assumed to transition from high-spin iron(II) to low-spin iron(III) upon O_2 binding.

We focused on Mn–porphyrin for our functional benchmarking study, as many functionals tested did not deliver the correct degree of electron transfer for the O_2-adsorbed structure, even after being initialized as Mn^{IV}–O_2^{2-}. As Table 20 indicates, TPSSh, PBE, and B97M-rV are the functionals that best capture a two-electron transfer to yield a peroxide species, and furthermore only TPSSh and B97M-rV provide reasonable estimates for the experimentally determined binding energy. Because no analytical Hessians were implemented for VV10 dispersion corrections in Q-Chem, obtaining vibrational frequency and entropy data would be more time consuming for B97M-rV. Thus, we calculated most of this data with TPSSh (see Tables S20 and S21 for calculated ΔS and O–O vibrational frequencies using TPSSh for Co-, Mn-, and Fe-PCN-224).

The entropy of O_2 binding for each cluster, which needs to account for additional configurations of the bound O_2 molecule, was computed as:

$$\Delta S_{ads} = \left(S_{total,f} + S_{conf,f}\right) - \left(S_{total,O2} + S_{total,i}\right) \tag{13}$$

$$S_{total,x} = S_{trans,x} + S_{rot,x} + S_{vib,x} \tag{14}$$

where $S_{total,x}$ is the entropy of a given system, x, i and f represent the initial and final state, respectively, and S_{conf}, S_{trans}, S_{rot}, and S_{vib} represent the configurational, translational, rotational, and vibrational contributions to the entropy. The contributions from configurational entropy can be estimated by $R\ln(N)$ where R is the gas constant and N is the number of configurations. Note that this assumes all N configurations are degenerate. For side-on O_2 binding geometry like that in Mn-PCN-224, N = 4 due to the fourfold degeneracy that can be intuited from the symmetry of the binding site and verified from the single-crystal x-ray diffraction structure of the O_2-bound structure. End-on binding geometry results in an 8-fold degeneracy, meaning N = 8 for Co-PCN-224 and Fe-PCN-224. Adding the fourfold configurational entropy to the calculated ΔS_{ads} for O_2 binding in Mn-PCN-224 with TPSSh, we obtain a final calculated binding entropy of −179 J mol^{-1} K^{-1}, which is similar to the value calculated in this work using the Clausius–Clapeyron relationship, −174 ± 20 J mol^{-1} K^{-1} (see Tables S20 and S11).

To calculate the configurational entropy contribution for end-on O_2 structures, like those observed for Co- and Fe-PCN-224, the rotation of the O_2 molecule needs to be considered. Here, we performed potential energy surface scans by fixing the torsion angle of N–M–O in 15°

increments for both the Co-porphyrin and Fe-porphyrin using TPSSh. Note that while our calculated binding energies using TPSSh are within 10 kJ/mol of the experimental ground-state O_2 binding enthalpy, we should not expect quantitatively accurate excited states, though we expect qualitative trends will hold (see Figure S8). If $kT >> V_{max}$,where V_{max} is the energy barrier to rotation, we may assume a free rotor approximation and compute the entropy contribution accordingly. If $kT << V_{max}$ we may assume the configurational entropy is roughly $R\ln\Omega$, where Ω is the number of energetic minima along the rotation path. For $kT \sim V_{max}$, we must treat the internal rotation rigorously as a hindered rotor. Interested readers may reference Pitzer and Gwinn's seminal work on this topic,[40] as well as challenges addressed by Pfaendtner et al.[41]

The Fe-porphyrin notably has a rotation barrier of approximately 5 kJ/mol with barriers at 90° increments (see Figure S8), meaning we may reasonably approximate the configurational entropy contribution as a 4-fold energetically degenerate contribution. Along with the additional 2-fold contribution of the O_2 binding with equal probability on the two sides of the porphyrin plane, this results in a total of eight degenerate positions. The Co-porphyrin, on the other hand, exhibits a binding energy corresponding to physisorption and accordingly we see a rotational barrier of 2 kJ/mol. The energetic minima, therefore, are accessible at finite temperature and would contribute greater configurational entropy than in the case of the Fe-porphyrin.

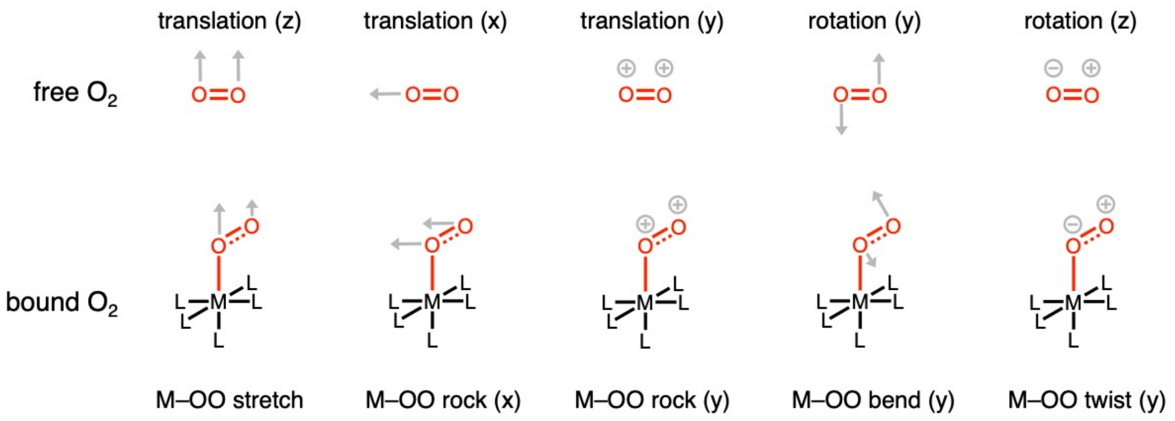

Figure S1. Correspondence between the degrees of freedom of free O_2 (top) and the vibrations of bound O_2 (bottom). Note that the M–OO twist shown at the bottom right of the figure should be treated as a free rotor when the barrier for rotation is small compared to k_BT.

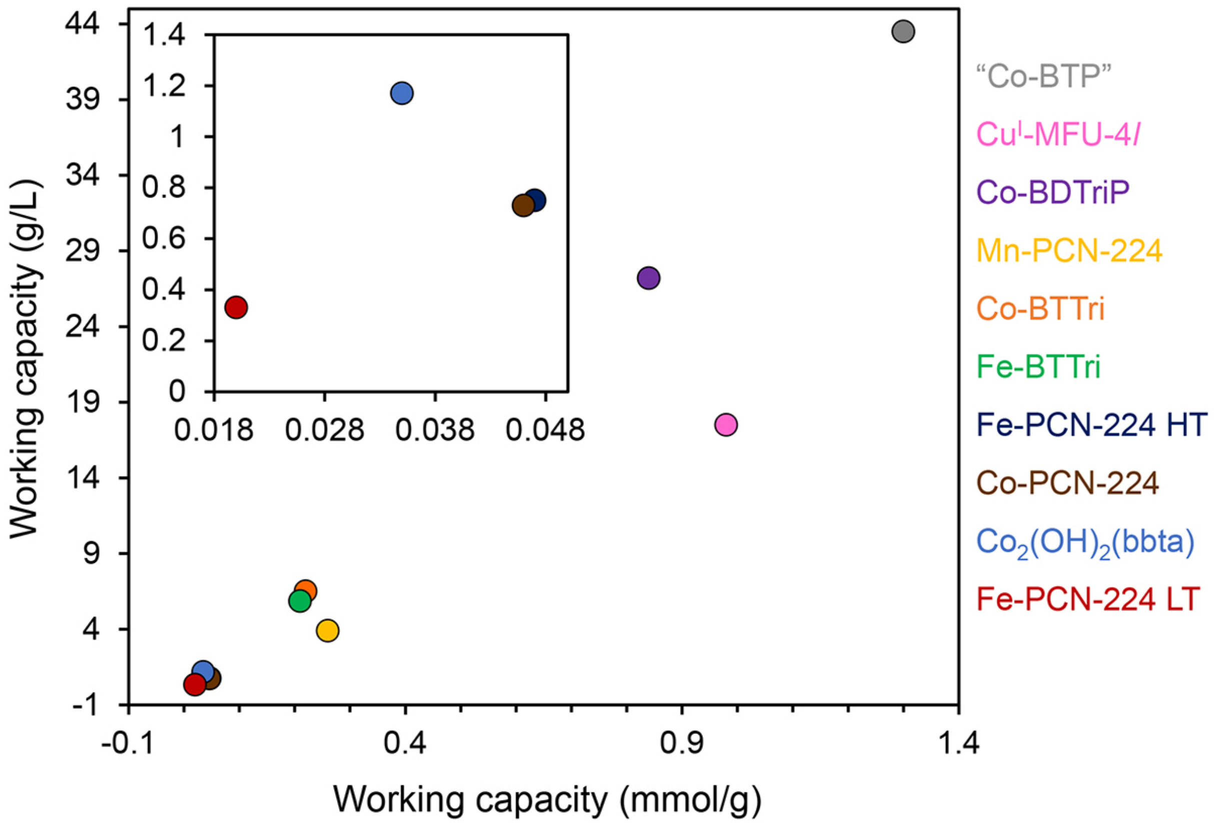

Figure S2. Calculated volumetric and gravimetric working capacities for select frameworks in a VSA process at 298 K involving adsorption of O_2 at 0.21 bar and desorption at 10 mbar. Values are tabulated in Table S14.

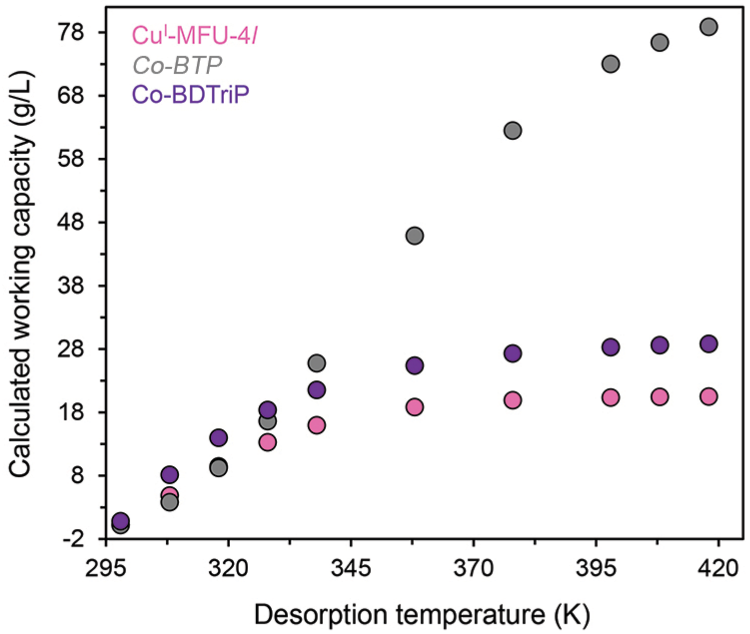

Figure S3. Calculated volumetric O_2 working capacities for Cu^{I}-MFU-4*l*, Co-BDTriP, and *Co-BTP* in a VTSA process with adsorption of air at 1 bar (0.21 bar O_2) and 298 K and desorption at 0.2 bar and varying temperatures. It is clear that the optimal VTSA conditions differ for each material, as a consequence of their differing free energy values for O_2 adsorption and volumetric metal site densities.

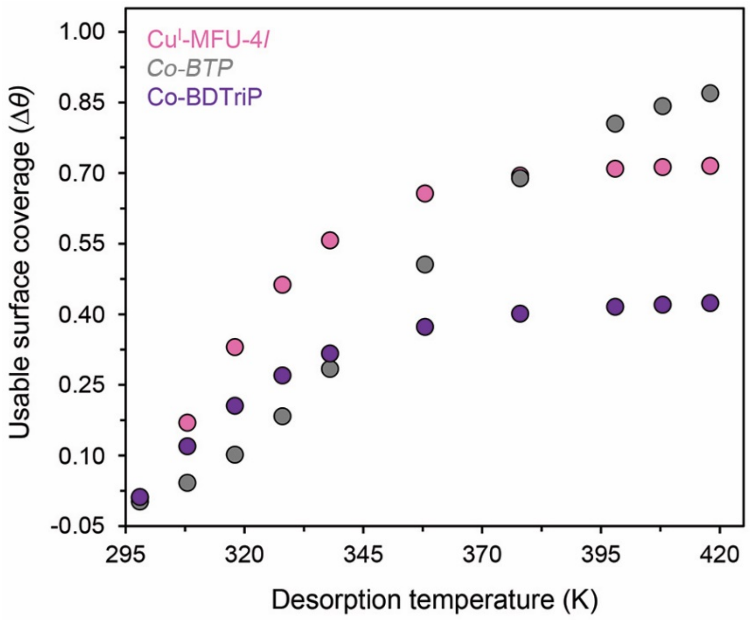

Figure S4. Calculated usable surface coverages ($\Delta\theta$) for Cu^{I}-MFU-4*l*, Co-BDTriP, and *Co-BTP* in a VTSA process with adsorption of O_2 at 0.21 bar and 298 K and desorption at 0.2 bar and varying temperatures. These three frameworks were chosen to illustrate different temperature-dependent behaviors given their relatively high usable surface coverages. The free energy values for O_2 adsorption have important consequences for optimal conditions. At 298 K, Co-BDTriP displays the highest useable coverage, at moderate-to-high temperatures, Cu^{I}-MFU-4*l* instead exhibits the highest values, while above 395 K, *Co-BTP* has the greatest $\Delta\theta$.

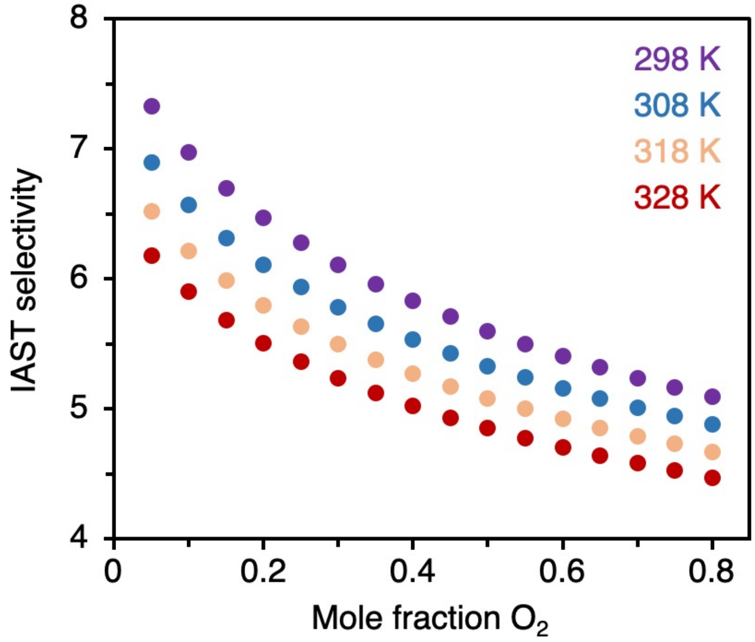

Figure S5. IAST O_2/N_2 selectivities for Cu^{I}-MFU-4*l* at four different temperatures for a 1 bar inlet feed of O_2 and N_2 in varying ratios. The dual-site Langmuir-Freundlich parameters used to obtain these values are given in Table S1. These calculations are based on experimental isotherms collected at low temperatures (203 to 233 K).[42]

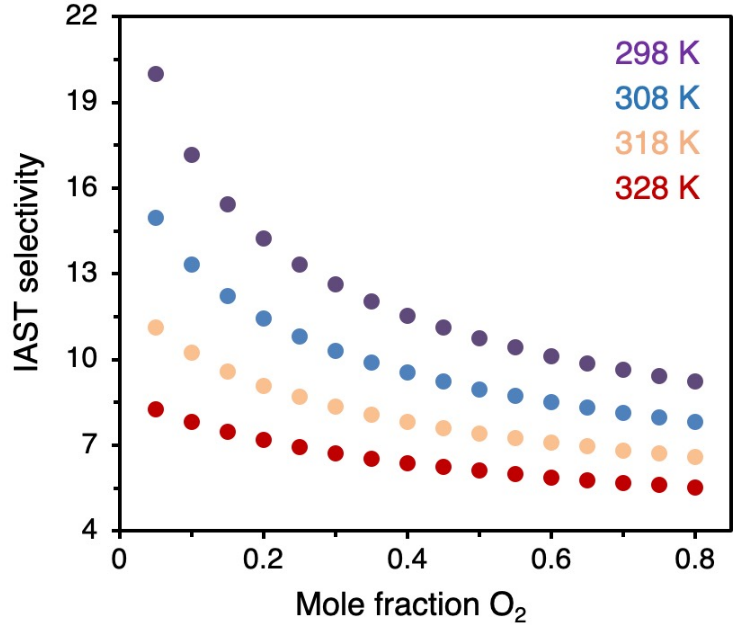

Figure S6. IAST O_2/N_2 selectivities for Co-BDTriP at the indicated temperatures for a 1 bar inlet feed of O_2/N_2 in varying ratios. The multi-site Langmuir-Freundlich parameters used to obtain these values were obtained from a previous report and the O_2 isotherm values are contained in Table S5. These calculations are based on experimental isotherms collected at low temperatures (195 to 226 K).[22]

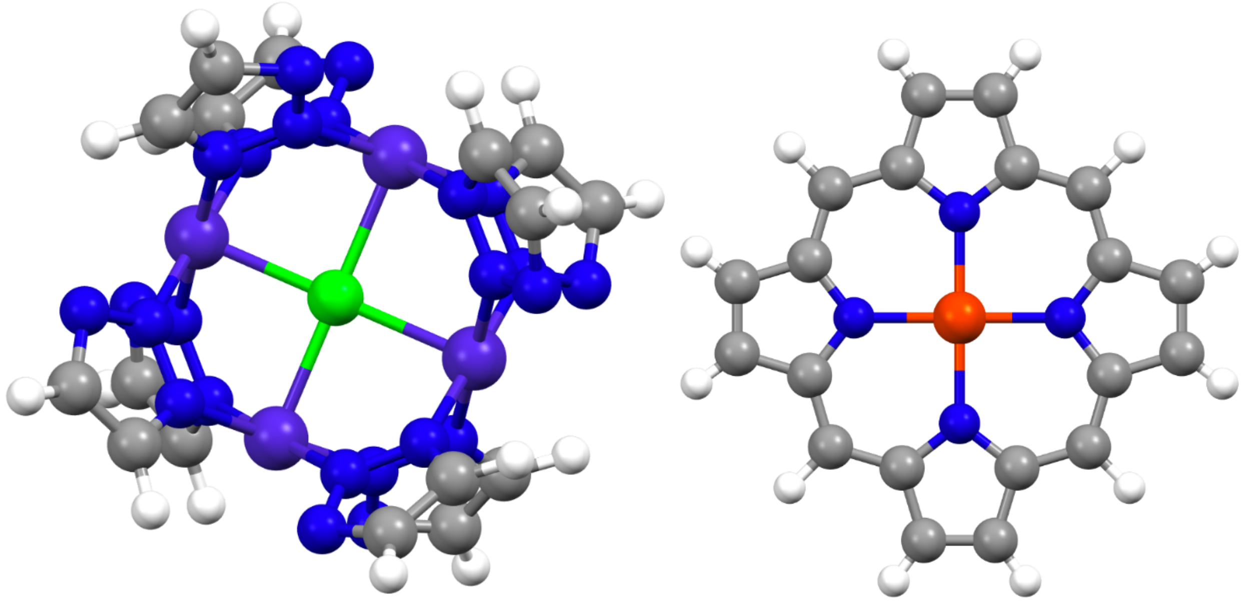

Figure S7. Representative structures for the $[M_4X(azolate)_8]^-$ and M-PCN-224 clusters.

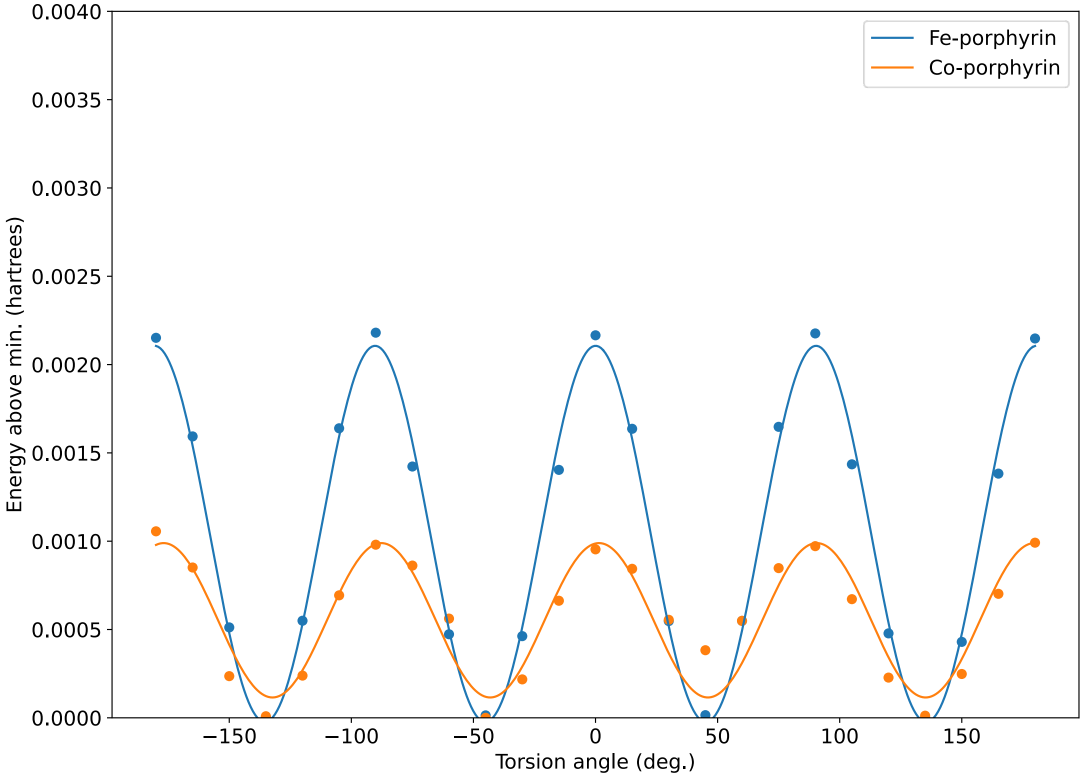

Figure S8. Potential energy surface scans of rotating around the metal–O bond in Fe– and Co–porphyrin. Points denote energies obtained via DFT cluster calculations using the methodology explained above. Lines represent curve fits to a single harmonic model of the form $b + a\cos(\phi + \frac{\pi f \theta}{180})$, where b is the bias term, a is the amplitude, ϕ is the phase shift, f is the frequency, and θ is the angle in degrees. The barrier to rotation for Co-porphyrin is roughly 1 millihartree (~2.6 kJ/mol) while the barrier to rotation for Fe-porphyrin is roughly 2 millihartree (~5.2 kJ/mol).

Table S1. Parameters obtained from fits to O_2 adsorption data for Cu^I-MFU-4*l* at the indicated temperatures, using a dual-site Langmuir-Freundlich model (eq. 7 and 8). Published isotherm data were newly fit in this work to obtain these parameters.[42]

Parameter	Values			
	**203 K**	**213 K**	**223 K**	**233 K**
$n_{sat,1}$ (mmol/g)	1.642	1.642	1.636	1.631
E_1 (−kJ/mol)	53.433	53.119	52.418	51.778
S_1 (−J/mol·K)	168.480	165.660	161.730	157.930
v_1	0.896	0.894	0.893	0.889
$n_{sat,2}$ (mmol/g)	4.810	4.809	4.870	4.883
E_2 (−kJ/mol)	16.348	16.330	16.311	16.344
S_2 (−J/mol·K)	87.418	86.403	85.379	84.453
v_2	0.907	0.937	0.946	0.9289

Table S2. Parameters obtained from fits to O_2 adsorption data for $Co_2(OH)_2$(bbta) at the indicated temperatures, using a dual-site Langmuir-Freundlich model (eq. 7 and 8). These parameters are reported directly from a previous publication.[43]

Parameter	Values		
	**195 K**	**213 K**	**223 K**
$n_{sat,1}$ (mmol/g)	2.457	2.457	2.457
E_1 (−kJ/mol)	49.836	50.982	50.082
S_1 (−J/mol·K)	199.00	203.05	200.19
v_1	1	1	1
$n_{sat,2}$ (mmol/g)	8.332	8.332	8.332
E_2 (−kJ/mol)	16.258	16.651	16.495
S_2 (−J/mol·K)	79.64	81.037	81.635
v_2	1	1	1

Table S3. Parameters obtained from fits to O_2 adsorption data for Fe-BTTri using a dual-site Langmuir-Freundlich model (eq. 7 and 8). These parameters were obtained directly from a previous publication,[24] where isotherms obtained at −78, −61, and −49 °C were simultaneously fit to one dual-site Langmuir-Freundlich equation.

Parameter	Values
$n_{sat,1}$ (mmol/g)	2.320
E_1 (−kJ/mol)	38.000
S_1 (−J/mol·K)	137.19
v_1	0.748
$n_{sat,2}$ (mmol/g)	7.290
E_2 (−kJ/mol)	12.100
S_2 (−J/mol·K)	62.940
v_2	1.0900

Table S4. Parameters obtained from fits to O_2 adsorption data for Co-BTTri using a dual-site Langmuir-Freundlich model (eq. 7 and 8). These parameters were obtained directly from a previous publication,[22] where isotherms obtained at 195, 213, and 223 K were simultaneously fit using one dual-site Langmuir-Freundlich equation.

Parameter	Values
$n_{sat,1}$ (mmol/g)	2.780
E_1 (−kJ/mol)	34.590
S_1 (−J/mol·K)	132.49
v_1	1
$n_{sat,2}$ (mmol/g)	7.050
E_2 (−kJ/mol)	5.180
S_2 (−J/mol·K)	33.960
v_2	1

Table S5. Parameters obtained from fits to O_2 adsorption data for Co-BDTriP, using a multi-site Langmuir-Freundlich equation, obtained directly from a previous publication.[22] Note that isotherm data obtained at 195, 213, and 223 K were simultaneously fit to derive the given parameters.

Parameter	Values			
	**Site 1**	**Site 2**	**Site 3**	**Site 4**
$n_{sat,1}$ (mmol/g)	0.250	0.800	1.080	6.750
E_1 (−kJ/mol)	47.03	36.23	32.36	11.34
S_1 (−J/mol·K)	124.62	110.960	120.60	61.81
v_1	1	1	1	1

Table S6. Parameters obtained from fitting O_2 adsorption data for Mn-PCN-224 obtained at the indicated temperatures, using a dual-site Langmuir-Freundlich model (eq. 7 and 8). Values are reported directly from a previous publication.[34]

Parameter	Values		
	**223 K**	**273 K**	**298 K**
$n_{sat,1}$ (mmol/g)	6.090	50.000	50.000
b_1 (bar^{-1})	0.139	0.0107	0.011
v_1	0.955	0.778	0.778
$n_{sat,2}$ (mmol/g)	0.512	0.363	0.363
b_2 (bar^{-1})	10078.856	10	10
v_2	1.146	0.980	0.980

Table S7. Parameters obtained from fitting O_2 adsorption data for Co-PCN-224 obtained at the indicated temperatures, using a dual-site Langmuir-Freundlich model (eq. 7 and 8). Values are reported directly from a previous publication.[35]

Parameter	Values			
	**113 K**	**141 K**	**156 K**	**195 K**
$n_{sat,1}$ (mmol/g)	0.470	0.120	0.16	0.410
b_1 (bar^{-1})	1.0 x 10^7	3.0 x 10^7	210	0.19
v_1	1.8	2.1	1.9	1
$n_{sat,2}$ (mmol/g)	36.8	36.8	36.8	36.8
b_2 (bar^{-1})	0.83	0.24	0.15	0.056
v_2	0.60	0.58	0.64	0.850

Table S8. Parameters obtained from fitting O_2 adsorption data obtained for Fe-PCN-224 in low- and high-temperature regimes as indicated, using a dual-site Langmuir-Freundlich equation (eq. 7 and 8). Parameters were calculated again for this work using published isotherm data.[36]

Parameter	Low-temperature values			High-temperature values		
	**141 K**	**156 K**	**195 K**	**226 K**	**273 K**	**298 K**
$n_{sat,1}$ (mmol/g)	0.503	0.629	0.637	0.583	0.911	1.054
E_1 (−kJ/mol)	35.880	24.084	26.899	28.785	30.072	28.574
S_1 (−J/mol·K)	67.61	117.110	114.090	114.190	114.090	114.090
v_1	1.836	0.485	0.717	0.845	0.879	0.763
$n_{sat,2}$ (mmol/g)	18.000	6.779	4.439	3.152	2.470	3.209
E_2 (−kJ/mol)	9.000	4.988	5.938	3.731	3.342	1.676
S_2 (−J/mol·K)	70.673	33.182	34.619	30.155	33.182	29.556
v_2	0.826	1.040	1.390	1.153	1.543	1.144

Table S9. Parameters obtained from fitting N_2 adsorption data for Cu^I-MFU-4*l* at the indicated temperatures, using a dual-site Langmuir-Freundlich equation (eq. 7 and 8). Reported isotherm data was used to obtain these parameters.[42]

Parameter	Values			
	**183 K**	**193 K**	**203 K**	**213 K**
$n_{sat,1}$ (mmol/g)	1.708	1.711	1.705	1.670
E_1 (−kJ/mol)	48.237	48.402	48.638	49.022
S_1 (−J/mol·K)	163.410	163.410	168.220	167.760
v_1	1.067	1.067	0.999	1.007
$n_{sat,2}$ (mmol/g)	4.774	4.766	4.946	7.282
E_2 (−kJ/mol)	15.680	15.781	15.781	15.074
S_2 (−J/mol·K)	84.080	84.080	84.314	84.309
v_2	1.1417	1.140	1.078	1.025

Table S10. Parameters obtained from fitting N_2 adsorption data for Li-LSX at the indicated temperatures, using a dual-site Langmuir-Freundlich equation (eq. 7 and 8). These parameters were obtained directly from a previous publication.[3]

Parameter	Values			
	**210 K**	**240 K**	**270 K**	**300 K**
$n_{sat,1}$ (mmol/g)	3.95	2.79	3.01	2.64
b_1 (Pa^{-1})	1.92×10^{-6}	1.61×10^{-6}	8.46×10^{-7}	5.62×10^{-7}
$n_{sat,2}$ (mmol/g)	2.54	2.21	2.00	1.70
b_2 (Pa^{-1})	3.01×10^{-4}	7.16×10^{-5}	2.14×10^{-5}	8.18×10^{-6}

Table S11. Calculated ΔH, ΔS, and ΔG_{298} values for O_2 binding to open-metal sites in selected frameworks as discussed in the main text. The Clausius–Clapeyron relationship (eq. 9, Section 5 above) was implemented with the isotherm fit data given in Tables S1 to S9 to obtain ΔH and ΔS, which were then used to calculate ΔG_{298}. In general, the temperatures used to calculate ΔH and ΔS are below room temperature, and we assume here that the ΔG at those temperatures is the same as at 298 K, ΔG_{298}. The results are ordered from most exothermic to least exothermic of O_2 binding. For more information, including low-loading values, please see the supporting Excel file, "O2IsothermFits". The values for *Co-BTP* were approximated from the highest O_2 binding enthalpy and entropy values for Co-BDTriP.

**MOF**	**ΔH (kJ/mol)**	**ΔS (J mol^{-1} K^{-1})**	**ΔG_{298} (kJ/mol)**
Mn-PCN-224	−56 ± 5	−174 ± 20	−4 ± 5
Cu^{I}-MFU-4*l*	−53 ± 1	−157 ± 3	−6 ± 1
Fe-BTTri	−50.5 ± 0.1	−175.2 ± 0.6	1.7 ± 0.1
$Co_2(OH)_2$bbta	−47 ± 2	−180 ± 9	6 ± 2
Co-BTP	*−46*	*−117*	*−11*
Co-BDTriP	−35.5 ± 0.1	−108.3 ± 0.5	−3.2 ± 0.1
Co-BTTri	−33.1 ± 0.3	−118 ± 1	1.9 ± 0.3
Fe-PCN-224 (LT)	−32 ± 5	−121 ± 30	4 ± 5
Co_2(dobdc)	−19	−55	−3
Fe-PCN-224 (HT)	−19 ± 2	−70 ± 10	2 ± 2
Co-PCN-224	−15.2 ± 0.4	−59.6 ± 0.7	2.6 ± 0.4

Table S12. Data used for calculating gravimetric and volumetric working capacities as reported in Table 5 in the main text and Tables S13 and S14 below. Theoretical O_2 capacities reported in mmol/g and g/L assume one O_2 bound per framework open metal site. Estimated O_2 capacity was determined as described in Section S5 above. In the case of *Co-BTP*, the same value as determined for Co-BTTri was used. For each framework, the ratio of the estimated to theoretical O_2 capacity in mmol/g was used as a multiplicative factor to determine the estimated O_2 capacity in g/L. The latter value was then multiplied by the surface coverage for a given set of conditions to determine the volumetric working capacity.

**Column 1**	**Column 2**	**Column 3**	**Column 4**	**Column 5**	**Column 6**	**Column 7**
**Framework**	**Molecular Weight (g/mol)**	**Crystallographic volumetric density (g/cm^3)**	**Theoretical O_2 capacity (mmol/g)**	**Estimated O_2 capacity (mmol/g)[b]**	**Theoretical O_2 capacity (g/L)**	**Estimated O_2 capacity (g/L)[d]**
Co-BTP	*6176.26*	*1.012*[a]	*3.89*	*2.8*[c]	*126.0*	*90.7*
Cu^I-MFU-4*l*	1186.77	0.560	1.69	1.6	30.3	28.7
Co-BDTriP	6207.88	1.012	3.87	2.1	125.3	68.0
Mn-PCN-224	4081.82	0.466	0.73	0.55	10.9	8.2
Co-BTTri	6223.69	0.919	3.86	2.8	113.5	82.3
Fe-BTTri	6140.31	0.883	3.91	2.3	110.5	65.0
Fe-PCN-224 HT	4084.54	0.498	0.73	0.55	11.6	8.8
Co-PCN-224	4093.8	0.493	0.73	0.70	11.5	11.0
$Co_2(OH)_2$(bbta)	310	1.046	6.45	2.5	215.9	83.7
Fe-PCN-224 LT	4084.54	0.498	0.73	0.55	11.6	8.8

[a] Assumed to be the same as Co-BDTriP.

[b] Estimated in most cases from the inflection point of the experimental isosteric heat of adsorption as a function of loading, which was approximated from the second derivative of the enthalpy versus loading, estimated using the finite difference method (see Section S5).

[c] Assumed to be the same as for Co-BTTri.

[d] Determined by multiplying the ratio of the Estimated and Theoretical Capacities in mmol/g by the Theoretical capacity in g/L, i.e., $\frac{\text{Column 5}}{\text{Column 4}} \times \text{Column 6}$.

Table S13. Calculated working capacities of various frameworks in a VSA process assuming desorption at 1 mbar, ordered by decreasing working capacity (mmol/g). In the case of Fe-PCN-224, the capacities at low temperature (LT) and high temperature (HT) were determined separately (see Table S8). The density of exposed metal sites in *Co-BTP* was is assumed to be equal to that in Co-BTTri.

P_{ads} = 1 bar (0.21 bar O_2), 298 K and P_{des} = 0.001 bar, 298 K					
Framework	Estimated O_2 capacity (mmol/g)	Estimated O_2 capacity (g/L)	Surface coverage ($\Delta\theta$)	Working capacity (mmol/g)	Working capacity (g/L)
Co-BTP	*2.8*	*90.7*	*0.87*	*2.4*	*78.9*
Cu^I-MFU-4*l*	1.6	28.7	0.71	1.1	20.4
Co-BDTriP	2.1	68.0	0.43	0.90	29.2
Mn-PCN-224	0.55	8.2	0.52	0.29	4.3
Co-BTTri	2.8	82.3	0.083	0.23	6.83
Fe-BTTri	2.3	65.0	0.095	0.22	6.2
Fe-PCN-224 HT	0.55	8.8	0.090	0.050	0.79
Co-PCN-224	0.70	11.0	0.069	0.048	0.76
$Co_2(OH)_2$(bbta)	2.5	83.7	0.014	0.035	1.17
Fe-PCN-224 LT	0.55	8.8	0.039	0.021	0.34

Table S14. Calculated working capacities of various frameworks in a VSA process assuming desorption at 10 mbar, ordered by decreasing working capacity (mmol/g). In the case of Fe-PCN-224, the capacities at low temperature (LT) and high temperature (HT) were determined separately (see Table S8). The density of exposed metal sites in *Co-BTP* was is assumed to be equal to that in Co-BTTri.

P_{ads} = 1 bar (0.21 bar O_2), 298 K and P_{des} = 0.01 bar, 298 K					
Framework	Estimated O_2 capacity (mmol/g)	Estimated O_2 capacity (g/L)	Δ Surface coverage ($\Delta\theta$)	Working capacity (mmol/g)	Working capacity (g/L)
Co-BTP	*2.8*	*90.7*	*0.48*	*1.3*	*43.5*
Cu^I-MFU-4*l*	1.6	28.7	0.61	0.98	17.5
Co-BDTriP	2.1	68.0	0.40	0.84	27.2
Mn-PCN-224	0.55	8.2	0.48	0.26	3.9
Co-BTTri	2.8	82.3	0.079	0.22	6.50
Fe-BTTri	2.3	65.0	0.090	0.21	5.85
Fe-PCN-224 HT	0.55	8.8	0.085	0.047	0.75
Co-PCN-224	0.70	11.0	0.066	0.046	0.73
$Co_2(OH)_2$(bbta)	2.5	83.7	0.014	0.035	1.17
Fe-PCN-224 LT	0.55	8.8	0.037	0.020	0.33

Table S15. DFT relaxed lattice constants with periodic boundary conditions of the unit cells of $[(M_4Cl)_3(BTT)_8]_2$ (M-BTT), $[(M_4Cl)_3(BTTri)_8]_2$ (M-BTTri), and the hypothetical $[(M_4Cl)_3(BTP)_8]_2$ (M-BTP) frameworks. Each unit cell is cubic and contains 12 metals from its three metal nodes.

Framework	Lattice Constant (Å)
Cr-BTT	18.9
Cr-BTTri	19.1
Cr-BTP	19.3
Mn-BTT	19.1
Mn-BTTri	19.3
Mn-BTP	19.6
Co-BTT	18.3
Co-BTTri	18.6
Co-BTP	18.8

Table S16. Results of DFT calculations with periodic boundary conditions on $[(M_4Cl)_3(BTT)_8]_2$ (M-BTT), $[(M_4Cl)_3(BTTri)_8]_2$ (M-BTTri), and the hypothetical $[(M_4Cl)_3(BTP)_8]_2$ (M-BTP) frameworks. The *U* values for Cr, Mn, and Co were 3.8, 3.5, and 3.3, respectively.

Framework	O_2 binding energy (kJ/mol)	*d*(M–O_2) bond (Å)	Spin density on M with O_2 bound / unbound (Bohr magneton)
Cr-BTT	58.9	2.01	3.41 / 3.78
Cr-BTTri	60.3	1.98	3.28 / 3.79
Cr-BTP	101.3	1.96	3.20 / 3.76
Mn-BTT	23.7	2.18	4.49 / 4.61
Mn-BTTri	28.6	2.15	4.38 / 4.59
Mn-BTP	40.8	2.16	4.31 / 4.58
Co-BTT	31.4	2.00	0.61 / 0.94
Co-BTTri	39.7	1.95	0.48 / 0.93
Co-BTP	53.3	1.92	0.34 / 0.92

Table S17. Results of DFT calculations with periodic boundary conditions on $[(Co_4X)_3(BTT)_8]_2$ (M-BTT-X), $[(M_4X)_3(BTTri)_8]_2$ (M-BTTri-X), and the hypothetical $[(Co_4X)_3(BTP)_8]_2$ (M-BTP-X) frameworks. *U* values for Cr, Mn, and Co were 3.8, 3.5, and 3.3, respectively.

Framework	O_2 binding energy (kJ/mol) X = F	O_2 binding energy (kJ/mol) X = Cl	O_2 binding energy (kJ/mol) X = Br	O_2 binding energy (kJ/mol) X = I
Co-BTT-X	25.1	31.4	31.7	38.6
Co-BTTri-X	33.3	39.7	45.5	53.7
Co-BTP-X	44.5	53.3		70.2

Table S18. Benchmarking functionals for the O_2 binding energy in Co-BTTri. The experimental isosteric heat of adsorption at low loading is approximately −34 kJ/mol (see Ref. 22).

**Functional**	**Binding energy (kJ/mol)**
MN15-L-D3(0)	−99
BP86-D3(BJ)	−84
TPSS-D3(BJ)	−78
M06-L-D3(0)	−73
revTPSSh-D3(BJ)	−48
TPSSh-D3(BJ)	−43
M06-D3(0)	−29
B3LYP-D3(BJ)	−26
M06-2X-D3(0)	−23
*ω*B97M-V	−23
*ω*B97X-D3	−15
PBE0-D3(BJ)	−15
*ω*B97X-V	−6.7
B97-2	−0.9

Table S19. DFT results on clusters for O_2 binding energies and metal–O_2 bond lengths. The functional TPSSh was used for Mn, Fe, and Co, and M06 was used for Cr.

Framework	Binding energy (kJ/mol)	d(M–O_2) bond (Å)
Cr-BTT	66	1.95
Cr-BTTri	79	1.98
Cr-BTP	89	1.96
Mn-BTT	28	2.18
Mn-BTTri	31	2.15
Mn-BTP	50	1.95
Co-BTT	42	2.00
Co-BTTri	43	1.95
Co-BTP	58	1.92

Table S20. Results of cluster calculations on a model Mn–porphyrin system, used to approximate Mn-PCN-224, with different functionals. As a reference, the reported Mn-PCN-224 O_2 binding enthalpy is −49.6(8) kJ/mol.[34] Enthalpy and entropy values calculated in this work using the Clausius–Clapeyron relationship (see Table S11) are −56(5) kJ/mol and −174(20) J mol^{-1} K^{-1}.

Functional	Binding energy (kJ/mol)	Binding entropy (J mol^{-1} K^{-1})	O–O stretch (cm^{-1})
TPSSH	−69	−179	1020
M06	6.2	−157	1213
B97M-rV	−66		1054
ωB97X-D	22	−176	1208
MN15	−7	−166	1229
MN15-L	−28	−144	1256
B3LYP	−14	−162	1100
PBE	−146	−162	1023
PBE0	29.5	−161	1349

Table S21. Results of cluster calculations for O_2 binding in Fe- and Co-PCN-224 using the TPSSh functional. Previously reported binding enthalpy values are included for comparison (refs. 36 and 35, respectively). Enthalpy and entropy values calculated in this work using the Clausius–Clapeyron relationship are given in Table S11.

Framework	Experimental binding enthalpy (kJ/mol)	Calculated binding energy (kJ/mol)	Calculated binding entropy (J mol^{-1} K^{-1})	Calculated O–O stretch (cm^{-1})
Fe-PCN-224	−34(4)	−29	−143	1291
Co-PCN-224	−15.2(6)	−27	−139	1417

References

1 A. M. Tsirlin and I. V. Titova, *Theor. Found. Chem. Eng.*, 2004, 38, 490–498.

2 B. A. Mersmann, B. Fill, R. Hartmann and S. Maurer, *Chem. Eng. Technol.*, 2000, **23**, 937–944.

3 D. Y. Koh, B. R. Pimentel, V. P. Babu, N. Stephenson, S. W. Chai, A. Rosinski and R. P. Lively, *Micropor. Mesopor. Mater.*, 2018, **256**, 140–146.

4 P. Atkins, J. de Paula, *Physical Chemistry*, W.H. Freeman and Company, 9th edn., 2010.

5 H. Swenson and N. P. Stadie, *Langmuir*, 2019, **35**, 5409–5426.

6 C. T. Campbell and J. R. V. Sellers, *Chem. Rev.*, 2013, **113**, 4106–4135.

7 R. T. Yang, in *Adsorbents: Fundamentals and Applications*, John Wiley & Sons, Inc., 2003, pp. 280–381.

8 A. S. Rosen, J. M. Notestein and R. Q. Snurr, *J. Chem. Phys.*, 2020, **152**, 224101.

9 R. Poloni, K. Lee, R. F. Berger, B. Smit and J. B. Neaton, *J. Phys. Chem. Lett.*, 2014, **5**, 861–865.

10 P. E. Blöchl, *Phys. Rev. B*, 1994, **50**, 17953–17979.

11 G. Kresse and D. Joubert, *Phys. Rev. B*, 1999, **59**, 1758–1775.

12 G. Kresse and J. Hafner, *Phys. Rev. B*, 1993, **47**, 558–561.

13 G. Kresse and J. Furthmuller, *Phys. Rev. B*, 1996, **54**, 11169–11186.

14 G. Kresse and J. Furthmüller, *Comput. Mater. Sci.*, 1996, **6**, 15–50.

15 G. Kresse and J. Hafner, *Phys. Rev. B*, 1994, **49**, 14251–14269.

16 S. Grimme, J. Antony, S. Ehrlich and H. Krieg, *J. Chem. Phys.*, 2010, **132**, 154104

17 S. Grimme, S. Ehrlich and L. Goerligk, *J. Comput. Chem.*, 2011, **32**, 1456-1465

18 O. Bengone, M. Alouani, P. Blochl and J. Hugel, *Phys. Rev. B,* 2000, **62**, 784–791.

19 A. Rohrbach, J. Hafner and G. Kresse, *J. Phys. Condens. Matter*, 2003, **15**, 979–996.

20 S. Dudarev and G. Botton, *Phys. Rev. B - Condens. Matter Mater. Phys.*, 1998, **57**, 1505–1509.

21 A. Jain, G. Hautier, S. P. Ong, C. J. Moore, C. C. Fischer, K. A. Persson and G. Ceder, *Phys. Rev. B*, 2011, **84**, 045115.

22 D. J. Xiao, M. I. Gonzalez, L. E. Darago, K. D. Vogiatzis, E. Haldoupis, L. Gagliardi and J. R. Long, *J. Am. Chem. Soc.*, 2016, **138**, 7161–7170.

23 E. D. Bloch, W. L. Queen, M. R. Hudson, J. A. Mason, D. J. Xiao, L. J. Murray, R. Flacau, C. M. Brown and J. R. Long, *Angew. Chem. Int. Ed.*, 2016, **55**, 8605–8609.

24 D. A. Reed, D. J. Xiao, H. Z. H. Jiang, K. Chakarawet, J. Oktawiec and J. R. Long, *Chem. Sci.*, 2020, **11**, 1698–1702.

25 Y. Zhao and D. G. Truhlar, *J. Chem. Phys.*, 2006, **125**, 194101.

26 P. Verma and D. G. Truhlar, *Trends Chem.*, 2020, **2**, 302–318.

27 Yumiao Ma, *ChemRxiv*, 2020, doi:10.26434/chemrxiv.11888838.v2

28 T. Weymuth, E. P. A. Couzijn, P. Chen and M. Reiher, *J. Chem. Theory Comput.*, 2014, **10**, 3092–3103.

29 K. A. Moltved and K. P. Kepp, *ChemPhysChem*, 2019, **20**, 3210–3220.

30 H. S. Yu, X. He, S. L. Li and D. G. Truhlar, *Chem. Sci.*, 2016, **7**, 5032–5051.

31 B. Chan, P. M. W. Gill and M. Kimura, *J. Chem. Theory Comput.*, 2019, **15**, 3610–3622.

32 K. A. Moltved and K. P. Kepp, *J. Chem. Theory Comput.*, 2018, **14**, 3479–3492.

33 K. Sumida, D. Stück, L. Mino, J. Da Chai, E. D. Bloch, O. Zavorotynska, L. J. Murray, M. Dincaì, S. Chavan, S. Bordiga, M. Head-Gordon and J. R. Long, *J. Am. Chem. Soc.*, 2013, **135**, 1083–

1091.

34 A. T. Gallagher, J. Y. Lee, V. Kathiresan, J. S. Anderson, B. M. Hoffman and T. D. Harris, *Chem. Sci.*, 2018, **9**, 1596–1603.

35 A. T. Gallagher, M. L. Kelty, J. G. Park, J. S. Anderson, J. A. Mason, J. P. S. Walsh, S. L. Collins and T. D. Harris, *Inorg. Chem. Front.*, 2016, **3**, 536–540.

36 J. S. Anderson, A. T. Gallagher, J. A. Mason and T. D. Harris, *J. Am. Chem. Soc.*, 2014, **136**, 16489–16492.

37 P. Rydberg and L. Olsen, *J. Phys. Chem. A*, 2009, **113**, 11949–11953.

38 S. P. de Visser and M. J. Stillman, *Int. J. Mol. Sci.*, 2016, **17**, 1–25.

39 Y. K. Natarajan Sathiyamoorthy Venkataramanan, Ambigapathy Suvitha, Hitoshi Nejo, Hiroshi Mizuseki, *Int. J. Quantum Chem.*, 2011, **110**, 2340–2351.

40 K. S. Pitzer and W. D. Gwinn, *J. Chem. Phys.*, 1942, **10**, 428–440.

41 J. Pfaendtner, X. Yu and L. J. Broadbelt, *Theor. Chem. Acc.*, 2007, **118**, 881–898.

42 D. Denysenko, M. Grzywa, J. Jelic, K. Reuter and D. Volkmer, *Angew. Chem. Int. Ed.*, 2014, **53**, 5832–5836.

43 J. Oktawiec, H. Z. H. Jiang, J. G. Vitillo, D. A. Reed, L. E. Darago, B. A. Trump, V. Bernales, H. Li, K. A. Colwell, H. Furukawa, C. M. Brown, L. Gagliardi and J. R. Long, *Nat. Commun.*, 2020, **11**, 3087.
